# Protein Language Models Identify Early Folding Residues as Critical Hotspots for Pathogenic Variants and Cancer Prognosis

**DOI:** 10.3390/ijms27198914

**Published:** 2026-10-07

**Authors:** Lisha Guo, Fuchong Yuan, Na Zhou, Minghui Zhao, Ruotong Liu, Ming Jing, K. Anton Feenstra, Qingzhen Hou

**Affiliations:** 1 Department of Biostatistics, School of Public Health, Cheeloo College of Medicine, Shandong University, Jinan 250100, China; 10431240264@stu.qlu.edu.cn (L.G.); 202416440@mail.sdu.edu.cn (F.Y.);; 2 National Institute of Health Data Science of China, Shandong University, Jinan 250100, China; 3 Key Laboratory of Computing Power Network and Information Security, Ministry of Education, Shandong Computer Science Center (National Supercomputer Center in Jinan), Qilu University of Technology (Shandong Academy of Sciences), Jinan 250300, China; 4 School of Data and Computer Science, Shandong Women’s University, Jinan 250300, China; 5 IBIVU–Center for Integrative Bioinformatics, Department of Computer Science, Vrije Universiteit Amsterdam, 1081HV Amsterdam, The Netherlands

**Keywords:** protein language model, early folding residues, structural vulnerability, pathogenic variation, cancer prognosis

## Abstract

Identifying the specific residues that initiate protein folding is critical for understanding protein stability and the mechanisms of misfolding-related diseases. However, the systematic, proteome-wide identification of these early folding residues (EFRs) has been hampered by both experimental and computational limitations. Here, we present LMEFold, a deep learning framework using protein language models (PLMs) to accurately predict EFRs from amino acid sequence alone. LMEFold outperforms existing sequence-based predictors on the Start2Fold benchmark (ROC AUC = 0.831), and its predicted scores show consistent associations with independent HDX-NMR protection-factor measurements, providing complementary biophysical evidence. Applying LMEFold to annotate over 6.6 million variants across diverse genomic datasets, we find that pathogenic mutations curated in ClinVar are significantly enriched within EFRs (~17%) compared to millions of population variants from large-scale cohorts including the UKB and gnomAD databases (~10%, *P* < 0.001). Critically, in the MSK-MET pan-cancer clinical cohort, EFR mutations are associated with severe functional disruption and significantly reduced overall patient survival. Structural analysis of hotspot cancer mutations, such as *TP53* p.Y163C, suggests a mechanism whereby these variants destabilize protein structure by disrupting the early folding core. Our work establishes EFRs as critical sites of pathogenic vulnerability and provides a powerful framework for the rapid identification of folding-centric prognostic markers using sequence only.

## 1. Introduction

While tens of thousands of disease-associated mutations have been cataloged in the human genome, their underlying molecular mechanisms often remain elusive [1,2,3]. A fundamental challenge is to decipher how single amino acid substitutions perturb the protein conformational landscape to drive pathogenesis [4,5]. Although protein misfolding is a well-established pathogenic driver, the specific sequence features that render proteins vulnerable to such perturbations are poorly understood [6,7,8]. The folding trajectory is often initiated by a small number of key residues, known as early folding residues (EFRs), which form stable nucleation centers that guide the global folding pathway [9,10,11]. Consequently, mutations within these critical EFRs are expected to be particularly disruptive, exerting their pathogenic effects by perturbing the initial folding process [12].

Systematically mapping EFRs at a proteome-wide scale remains a significant bottleneck. Although experimental techniques such as hydrogen-deuterium exchange (HDX) mass spectrometry and nuclear magnetic resonance (NMR) provide high-resolution insights into folding dynamics, their low throughput and technical complexity limit large-scale application [13,14,15]. This limitation has motivated the development of computational predictors [16]. However, existing methods largely rely on handcrafted biochemical features, such as hydrophobicity and secondary structure propensity, which may not fully capture the global sequence context governing folding pathways [17]. As a result, their predictive performance and generalization remain limited, highlighting the need for models that can learn folding-relevant representations directly from sequence [18].

The recent emergence of large protein language models (PLMs) offers a powerful solution to this challenge [19]. Trained on billions of sequences, these models learn the fundamental “grammar” of the protein universe, capturing evolutionary constraints and physicochemical properties that enable inference of structural features directly from sequence [20,21]. Recent studies demonstrate that this extensive prior knowledge enables efficient adaptation on downstream tasks, such as stability and variant prediction, even when supervised training data is scarce [22]. These properties are particularly advantageous for modeling early folding signals, where experimental annotations are limited. Crucially, the underlying Transformer architecture captures the complex, long-range dependencies governing folding, overcoming the limitations of local, feature-based approaches. These capabilities make PLMs a particularly suitable foundation upon which to build an accurate and robust predictor for EFRs.

Building on this foundation, we present LMEFold, a deep learning framework based on the ESM-2 protein language model, to predict EFRs directly from sequence. We show that LMEFold outperforms the leading sequence-based predictor, EFoldMine [16], on independent test sets. Importantly, LMEFold identifies early folding signals that cannot be fully captured by structural confidence. By recovering a subset of true EFRs missed by AlphaFold’s pLDDT metric, LMEFold achieves higher discriminatory performance for this specific task (area under the receiver operating characteristic curve (AUC): 0.829 vs. 0.765). Leveraging its scalability, we systematically investigate the role of EFRs across large-scale genomic datasets, encompassing over 6.6 million variants from large-scale population and clinical cohorts. We find that EFRs are significantly enriched for pathogenic mutations across these datasets. Furthermore, in a large pan-cancer cohort, we reveal that mutations within EFRs are associated with significantly poorer patient survival. Through structural analysis of hotspot driver mutations, such as *TP53* p.Y163C, we illustrate the molecular mechanism by which these variants disrupt the early folding core to trigger functional loss. Together, these results establish EFRs as critical determinants of mutational vulnerability and provide a framework for linking protein sequence to disease-relevant functional outcomes, with potential applications in prioritizing variants of uncertain significance (VUS) at scale.

## 2. Results

### 2.1. Deciphering the Sequence Determinants of Protein-Folding Initiation

**Figure 1:**
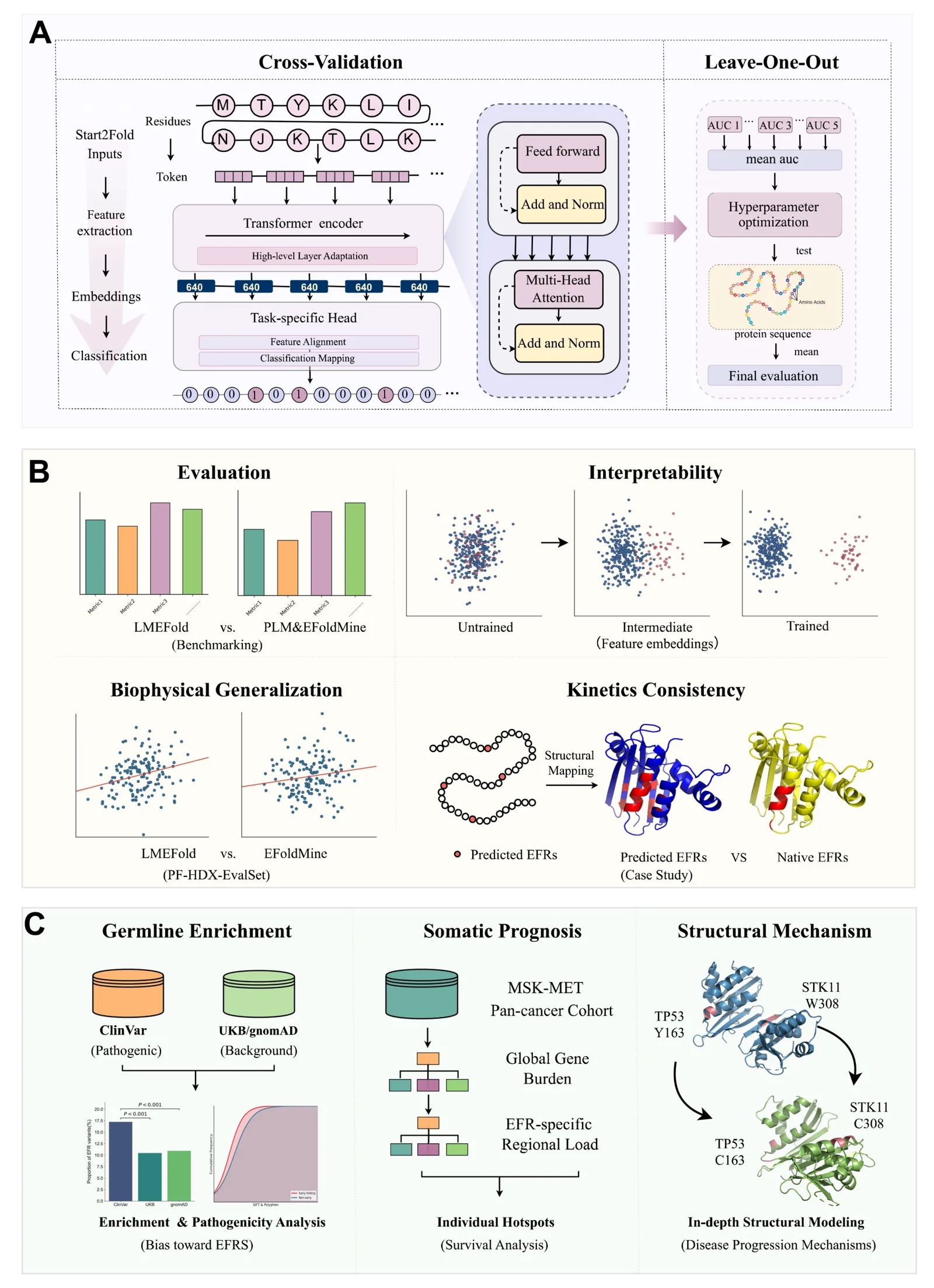
Overview of the LMEFold framework for identifying early folding residues as structurally vulnerable sites and linking them to disease mechanisms and clinical outcomes. (**A**) Early folding residue prediction model based on ESM-2 embeddings and a nested cross-validation training scheme. (**B**) Comprehensive performance evaluation, including benchmarking, interpretability analysis via t-SNE visualization, structural consistency checks (Case Study), and external biophysical validation on the PF-HDX-EvalSet. (**C**) Integrative clinical-structural analysis workflow linking early folding residues with germline pathogenic enrichment, somatic prognostic stratification, and in-depth structural modeling of disease-associated hotspots (e.g., *TP53* and *STK11*).

To systematically decode the role of early folding in disease, we established LMEFold, an integrative analytical framework that combines deep learning with biophysical validation and clinical interpretation (Figure 1).

The workflow begins with model construction (Figure 1A). In this module, sequence-level embeddings were derived from pre-trained PLMs to capture contextual dependencies and latent evolutionary constraints. These feature vectors were fed into a classifier incorporating dropout regularization and fully connected layers, projecting the representations into a binary classification space. Finally, a sigmoid activation function was applied to output the probability of each residue serving as an early folding site.

Following model development, we implemented a multi-faceted evaluation strategy to systematically assess model performance, generalization ability, interpretability, and structural consistency (Figure 1B). First, predictive performance was benchmarked against representative PLMs and the leading sequence-based predictor, EFoldMine, on standardized prediction tasks. To further examine generalization beyond the training distribution, we evaluated the model on independent biophysical data by correlating predicted scores with experimental log(PF) values (PF: ratio of intrinsic to experimental exchange rates; higher log(PF) reflects stronger structural protection) on the PF-HDX-EvalSet [23], thereby assessing the model’s ability to capture intrinsic structural stability signals associated with EFRs. In parallel, to interpret the learned representations, we employed t-SNE to visualize the evolution of feature embeddings across training stages, revealing the progressive separation of early folding and non-EFRs. Additionally, to examine structural consistency, we mapped predicted early folding probabilities onto the three-dimensional structures of representative proteins, including myoglobin (PDB: 1MBC) and ubiquitin (PDB: 1UBQ), enabling qualitative comparison with known folding kinetics and spatial organization.

Finally, to decipher the functional and clinical significance of EFRs, we integrated germline variant data (ClinVar [24], gnomAD [25], UK Biobank [26,27]) and somatic mutation profiles (MSK-MET [28]) to systematically investigate the association between early folding sites and human diseases (Figure 1C). First, using population variations from gnomAD and UK Biobank (UKB) as a background, we performed enrichment analysis to test whether pathogenic germline variants in ClinVar exhibit a significant distribution bias toward EFRs. Building on this, we leveraged the MSK-MET pan-cancer cohort to dissect the prognostic impact of somatic mutations across three distinct resolutions, ranging from global gene-level burden and EFR-specific regional load to individual pathogenic hotspots. Complementing these survival analyses, we performed in-depth structural modeling of variants such as *TP53* p.Y163C and *STK11* p.W308C to illustrate the potential mechanisms by which they regulate disease progression. This framework enables systematic identification, validation, and clinical interpretation of EFRs across the human proteome.

### 2.2. Model Optimization and Performance Evaluation

**Figure 2:**
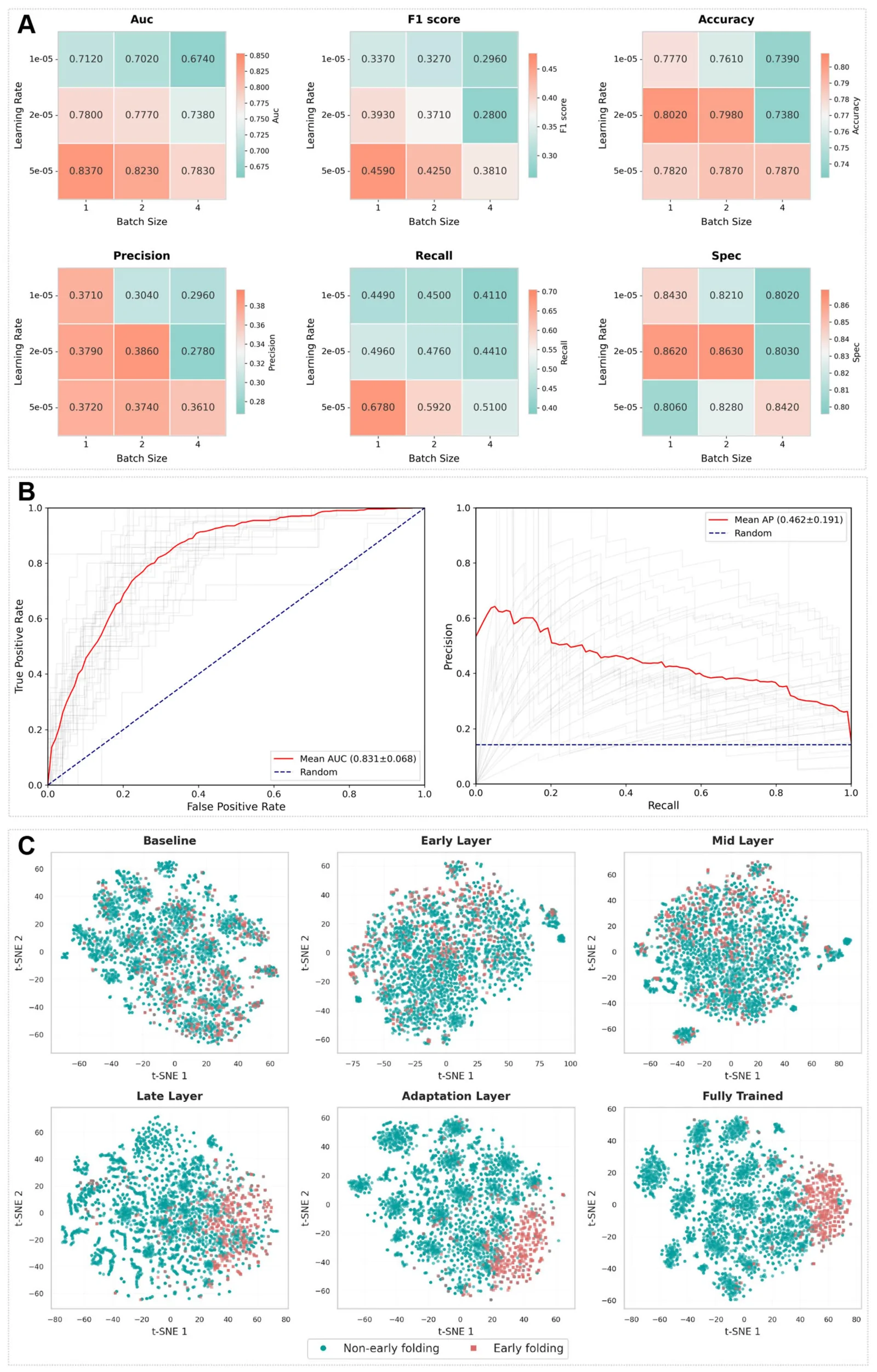
Hyperparameter optimization and performance evaluation of LMEFold. (**A**) Hyperparameter sensitivity analysis on the representative training case 9PCY. Heatmaps illustrate the impact of varying learning rate and batch size on six key performance metrics, identifying the optimal configuration for model convergence. Color gradients indicate metric values, from low (cyan) to high (red). (**B**) Generalization performance on the Benchmark30 dataset: ROC and PR curves obtained from leave-one-out cross-validation, where gray lines represent individual protein results, the solid red line denotes the mean performance, and the blue dashed line indicates the random baseline. (**C**) t-SNE visualization of the feature learning process, showing the progressive separation of early-folding residues (red) and non-early-folding residues (cyan) during training.

To address the challenges of limited sample size and severe class imbalance in early folding residue prediction, we systematically evaluated the training strategies and model performance. First, to ensure model stability under data-scarce conditions, we conducted hyperparameter optimization (Table 1). The results indicate that learning rate and batch size are the primary factors influencing model performance (Figure 2A and Table S5), whereas the effect of dropout rate is comparatively minor. Second, through a series of experiments with different class weight configurations, we quantitatively assessed the model’s robustness to class imbalance. As shown in Table S6, model performance exhibits only limited fluctuations across different weight settings, with the optimal performance achieved when the positive class weight is set to 5. This observation demonstrates that LMEFold is relatively insensitive to class weight variations and exhibits strong robustness against data imbalance. Under the optimal configuration, we further evaluated the model’s generalization ability under protein-level distribution shift using 27-fold leave-one-out cross-validation (LOOCV) with homology constraints on the Benchmark30 dataset. The results show that the model achieves an average ROC-AUC of 0.831 with a standard deviation of 0.068 (Figure 2B), indicating consistent predictive performance across different proteins and strong cross-sample stability. In addition, we employed the precision-recall (PR) curve as a complementary evaluation metric (Figure 2B). LMEFold achieves a PR-AUC of 0.462, demonstrating a favorable balance between precision and recall under imbalanced data conditions, and effective identification of minority (early folding) residues. Based on the Benchmark30 dataset (Table S1), we further conducted comparative experiments between LMEFold (Table S7) and several mainstream protein pretrained language models (Table 2), including ESM-2 (t12) (Table S8), ProtBERT (Table S9), and ProtT5 (Table S10). LMEFold achieves an AUC of 0.831, outperforming ProtBERT (0.800), ProtT5 (0.804), and ESM-2 t12 (0.812). These results indicate that, compared with general-purpose PLMs, LMEFold is more effective in capturing signals associated with early folding events. Furthermore, we performed a direct comparison with EFoldMine, which is currently the only dedicated tool for residue-level early folding site prediction and serves as a widely recognized benchmark in this field. LMEFold achieves a higher AUC (0.831) than EFoldMine (0.805, Table S11), and also outperforms it in terms of precision and specificity (with a DeLong test *P* = 0.014 confirming statistical significance), further confirming the reliability and superiority of our model for early folding residue prediction.

### 2.3. External Generalization Validation on Independent PF-HDX Datasets

To evaluate whether LMEFold generalizes beyond the Benchmark30 training dataset, we compared its performance with EFoldMine on the independent PF-HDX-EvalSet dataset, which was not used during model development or hyperparameter optimization. To further eliminate potential information leakage, a rigorous leave-one-out validation strategy was applied to proteins sharing >25% sequence identity between the training set and the external dataset. Specifically, for each overlapping protein, predictions were generated using a model retrained after excluding that protein from the training data, thereby ensuring that each target protein remained unseen during model training. Model performance was evaluated using the residue-level Pearson correlation coefficient (r), which quantifies the agreement between predicted early-folding scores and experimentally measured log(PF) values. As shown in Table S4, LMEFold consistently outperformed the baseline model, achieving a higher mean per-protein correlation (0.306 vs. 0.207) as well as a higher pooled correlation across all residues (0.168 vs. 0.106).

The moderate absolute values of these correlations are consistent with the biophysical distinction between the two metrics. While log(PF) primarily reflects thermodynamic equilibrium stability governed by the consolidation of the hydrophobic core, the early-folding score specifically characterizes the transient kinetic propensity of residues to initiate folding. Since the transition from kinetic initiation to the stabilized native state involves intricate conformational remodeling, a moderate correlation is mechanistically anticipated. Despite these inherent complexities, LMEFold correctly identifies the trend where high-PF regions correspond to early-folding residues in 90% (9/10) of the tested proteins, with the sole exception of 1TM1. This performance substantially exceeds that of EFoldMine and reinforces the biological rationality of our PLM-based framework. Together, these results demonstrate that LMEFold accurately captures early folding signals and generalizes across both sequence-based benchmarks and independent biophysical datasets.

### 2.4. Interpretability Study of Feature Representation Evolution

To elucidate the feature learning mechanism of LMEFold, we employed t-SNE to project high-dimensional representations into a two-dimensional space. Using the 9PCY training set as a representative case, we visualized the latent features across progressive network layers to trace the dynamic evolution of the model’s discriminative capacity for distinguishing between EFRs and non-EFRs (Figure 2C).

The visualizations across the training trajectory of 9PCY reveal a clear transition in feature distribution. At the initial baseline stage, EFRs (red) and non-EFRs (cyan) are extensively intermixed, reflecting an absence of learned discriminative information. As the network parameters were optimized through the intermediate layers (Early, Mid, and Late Layers), a distinct separation trend began to emerge. By the fully trained phase, the two classes converged into topologically distinct regions with significantly enhanced separability; this transition demonstrates that the model successfully extracted highly discriminative semantic features from the underlying protein sequences. These results indicate that LMEFold progressively abstracts informative representations throughout the learning process, which allows it to capture critical structural and evolutionary cues necessary for the accurate identification of early folding sites. These findings indicate that LMEFold learns biologically meaningful representations that distinguish folding-relevant residues from background sequence context.

### 2.5. Case Studies

To assess the biological relevance and mechanistic consistency of LMEFold in realistic biological systems, we selected myoglobin (PDB: 1MBC) and ubiquitin (PDB: 1UBQ) as joint case studies. Both proteins are supported by millisecond-timescale, residue-resolved early-folding HDX-NMR measurements, yet they differ substantially in molecular size, structural composition, and the coupling between folding and native-state stability. Notably, myoglobin provides both early-folding and native-state stability annotations, whereas ubiquitin offers early-folding annotations only and lacks any cofactor dependence. This complementary pairing enables a stringent evaluation of whether the model genuinely captures folding-initiation nucleation features, rather than static structural stability or function-related signals.

**Figure 3:**
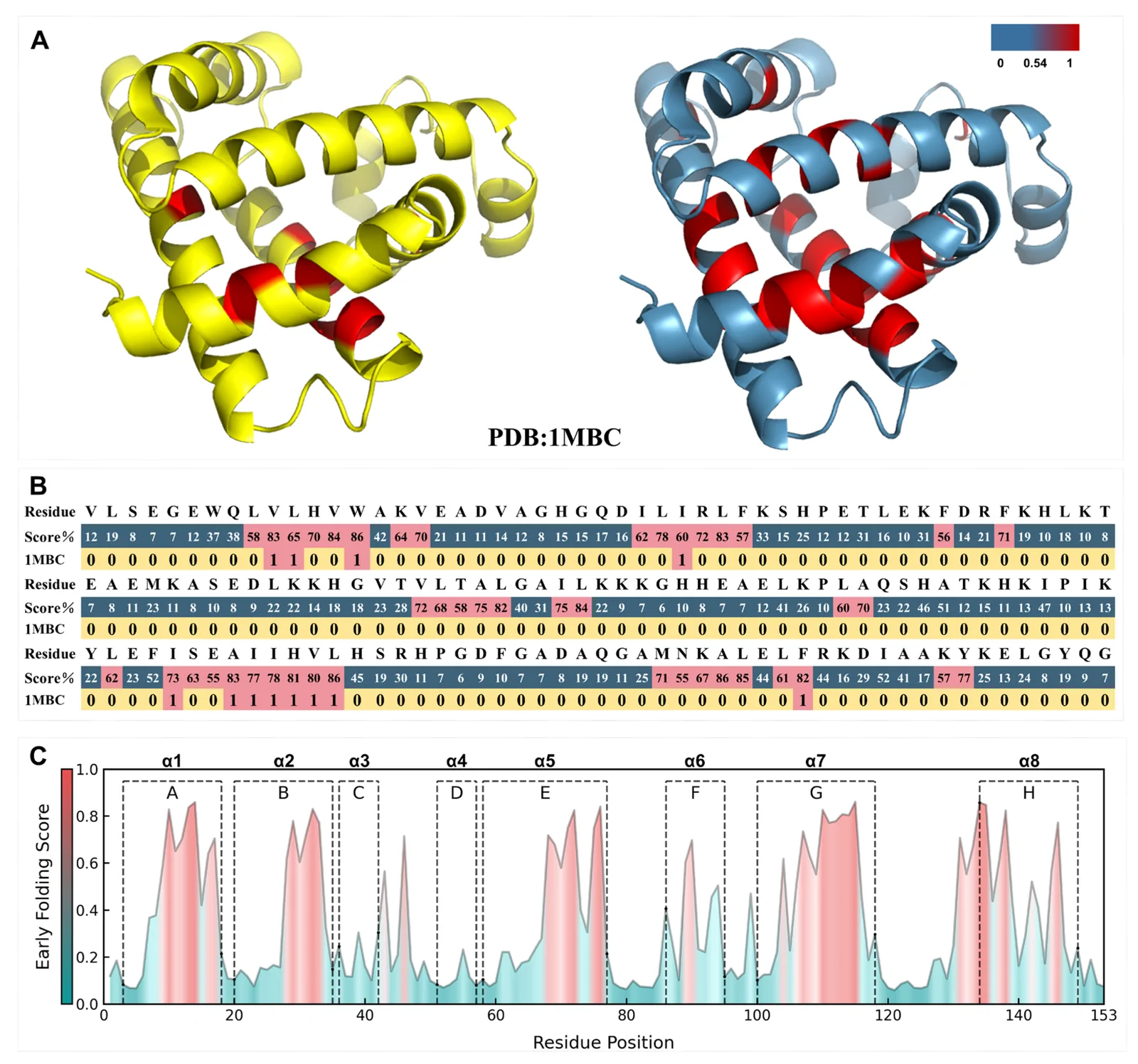
Early folding signals recapitulate experimentally defined nucleation regions and structural organization in myoglobin (PDB: 1MBC). (**A**) Spatial distribution of experimentally identified early-folding residues mapped onto the three-dimensional structure of myoglobin (left, red), alongside the structural mapping of LMEFold-predicted early-folding probabilities (right; color scale from blue to red indicates increasing probability). (**B**) Residue-level alignment of predictions versus experimental annotations. The top row displays the amino acid sequence; the middle row presents the LMEFold-predicted probabilities (ranging from 0 to 100%), with red cells indicating residues predicted as early-folding sites; the bottom row shows the binary experimental labels (1 = early folding, red; 0 = non-early folding, yellow). (**C**) Distribution of early-folding scores across the protein sequence. The continuous curve represents predicted probabilities, with a color gradient from blue (low score) to red (high score). Annotated boxes (**A**–**H**) correspond to the eight canonical α-helical segments, demonstrating the consistency between high-scoring regions and secondary structure elements.

In myoglobin, high-probability regions predicted by LMEFold cover the majority of experimentally identified early-folding residues and show strong spatial overlap with several known α-helical segments (Figure 3). This pattern is consistent with a nucleation mechanism driven by the preferential formation of local secondary structure elements, which subsequently establish the global folding topology. These results indicate that LMEFold can effectively identify key structural units involved in the initial folding stage, even in systems where cofactors are present and folding–stability contributions are experimentally separable.

For ubiquitin, despite its highly compact structure and a folding process that is largely governed by intrinsic sequence information, LMEFold achieves an AUC of 0.861. High-scoring regions similarly coincide with annotated α-helical elements and their neighboring structural units (Figure S1), demonstrating that the model can robustly capture sequence-encoded early-folding signals under conditions where no native-state stability reference is available and only early-folding experimental annotations are provided.

Taken together, these case studies show that LMEFold exhibits consistent and robust discriminative performance across proteins with distinct structural features and experimental constraints. The model reliably identifies early-folding nucleation residues, indicating that it captures signals aligned with the kinetic processes of the initial folding stage, rather than static structural properties or function-related features.

### 2.6. Pathogenicity and Clinical Implications of Early Folding Site Mutations

#### 2.6.1. Quantitative Enrichment and Functional Polarization of Pathogenic Mutations

**Figure 4:**
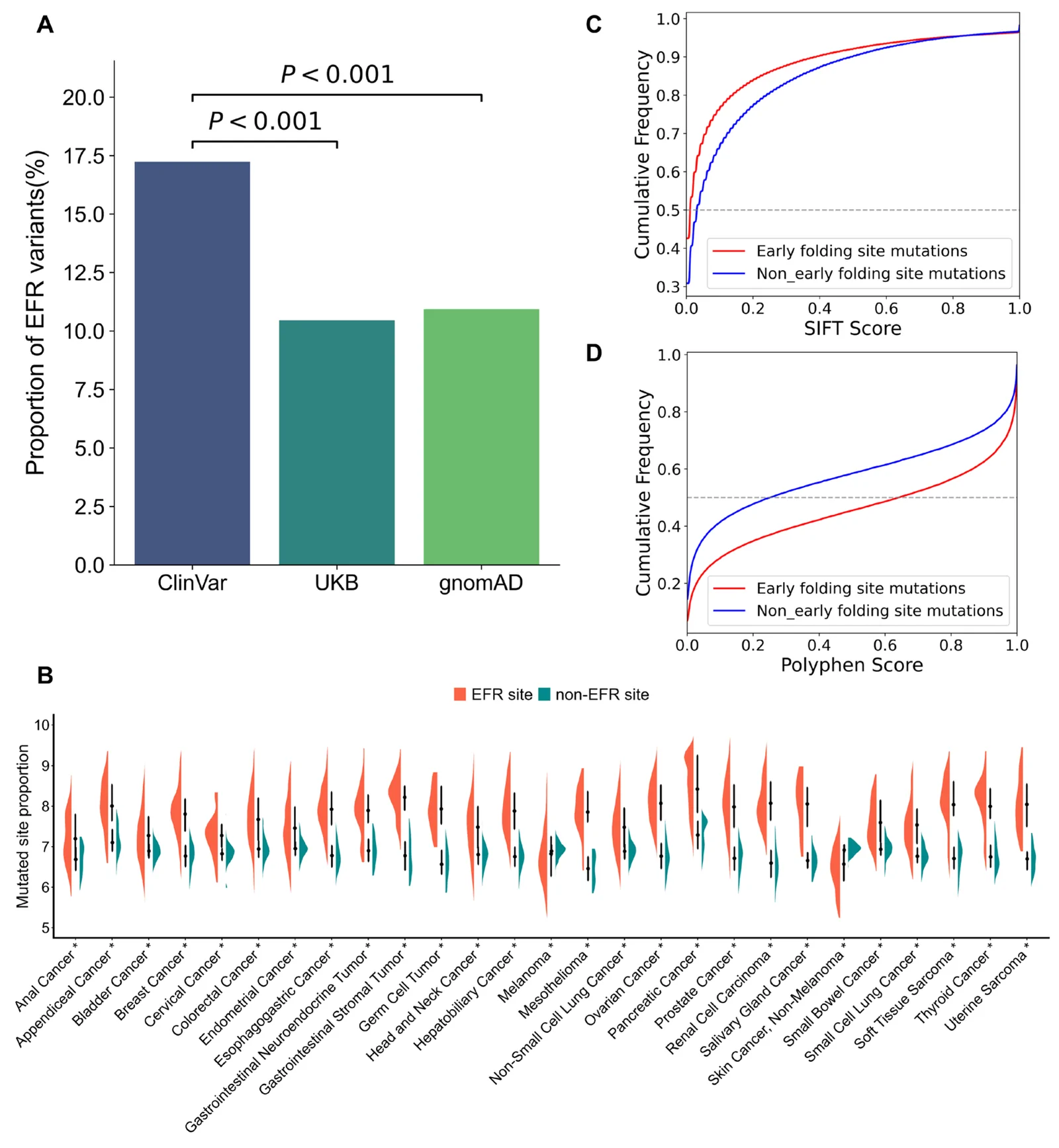
Early folding residues define structurally vulnerable regions enriched for pathogenic mutations. (**A**) Quantitative enrichment of pathogenic variants. The bar chart compares the proportion of variants located at early folding residues (EFRs) across ClinVar (pathogenic), UKB, and gnomAD (population controls). Statistical significance was assessed using the Mann–Whitney U test (*P* < 0.001). (**B**) Pan-cancer mutational burden analysis in the MSK-MET cohort. Violin plots display the proportion of mutated sites falling within EFRs (orange) versus non-EFRs (teal) across 27 cancer types. Statistical significance was evaluated using the Wilcoxon rank-sum test (* denotes *P* < 0.05). (**C**,**D**) Functional impact assessment using in-silico prediction tools. Cumulative distribution functions (CDF) are shown for (**C**) SIFT scores (where lower scores indicate higher deleteriousness) and (**D**) PolyPhen-2 scores (where higher scores indicate higher pathogenicity). Red curves represent mutations within EFRs, while blue curves represent non-EFR mutations, demonstrating a consistent shift toward higher predicted pathogenicity for EFR-resident variants.

The biological significance of EFRs is validated by a pronounced non-random distribution of disease-associated variants across the proteome, encompassing both germline and somatic contexts. First, in the germline context, statistical analysis revealed that the density of ClinVar pathogenic mutations within EFRs is approximately 1.7-fold higher than the baseline observed in gnomAD and UKB control populations (17% vs. 10%, *P* < 0.001; Figure 4A). Specifically, the odds ratio (OR) for ClinVar mutations compared to the UKB baseline is 1.78 (95% CI: 1.72–1.85), and the OR compared to the gnomAD baseline is 1.70 (95% CI: 1.64–1.76). Fine-grained analysis of ClinVar variants further confirmed these findings: 17.3%, 17.5%, and 15.8% of Pathogenic, Likely pathogenic, and Pathogenic/Likely pathogenic variants were located within EFRs, respectively (Figure S3). This enrichment indicates that although EFRs comprise only a fraction of the total protein sequence, they harbor a disproportionate share of the germline mutational burden associated with human pathology.

We next investigated whether this pattern persists in the somatic mutational landscape. Using the MSK-MET pan-cancer cohort, which encompasses 27 distinct cancer types, we observed a similar trend (Figure 4B). Notably, 25 cancers (92.6%) exhibited a significantly elevated proportion of somatic EFR mutations (Median enrichment scores > 1, *P* < 0.01). The only two exceptions showing significant depletion were non-melanoma skin cancer and melanoma (Median enrichment scores < 1), with full statistical breakdowns for each cancer type listed in Supplementary Table S12. This pervasive trend across diverse malignancies strongly reinforces the broad clinical relevance of the EFR enrichment pattern.

To further evaluate the functional impact of these mutations, variants were classified as “EFR mutations” or “non-EFR mutations” based on LMEFold predictions. The pathogenicity of these two mutation categories was then analyzed using SIFT scores (where closer to 0 indicates greater deleteriousness) [29] and PolyPhen-2 scores (where closer to 1 indicates greater deleteriousness) [30] derived from the VEP tool [31]. The results demonstrated that for SIFT scores, EFR mutations exhibited a higher cumulative frequency in the low-score range (closer to 0), signifying they are more frequently predicted as deleterious (Figure 4C). Similarly, for PolyPhen-2 scores, EFR mutations showed a higher cumulative frequency in the high-score range, indicating their greater potential to disrupt protein function (Figure 4D). The concordance between these two independent scoring systems reinforces that EFR mutations are more likely to negatively impact protein conformation and biological activity than those in non-EFR regions.

Collectively, by integrating germline and somatic distribution statistics with functional score analyses, we conclude that early folding sites are not only enriched for pathogenic variants but also represent vulnerable structural hotspots where mutations possess a heightened capacity to disrupt protein function. This finding highlights the critical role of folding initiation regions in the molecular pathogenesis of human disease. These results collectively support the view that early folding residues define structurally vulnerable regions that are preferentially targeted by pathogenic mutations.

#### 2.6.2. From Structural Damage to Clinical Outcome: Prognostic Impact of TP53 and STK11 Early Folding Site Mutations

**Figure 5:**
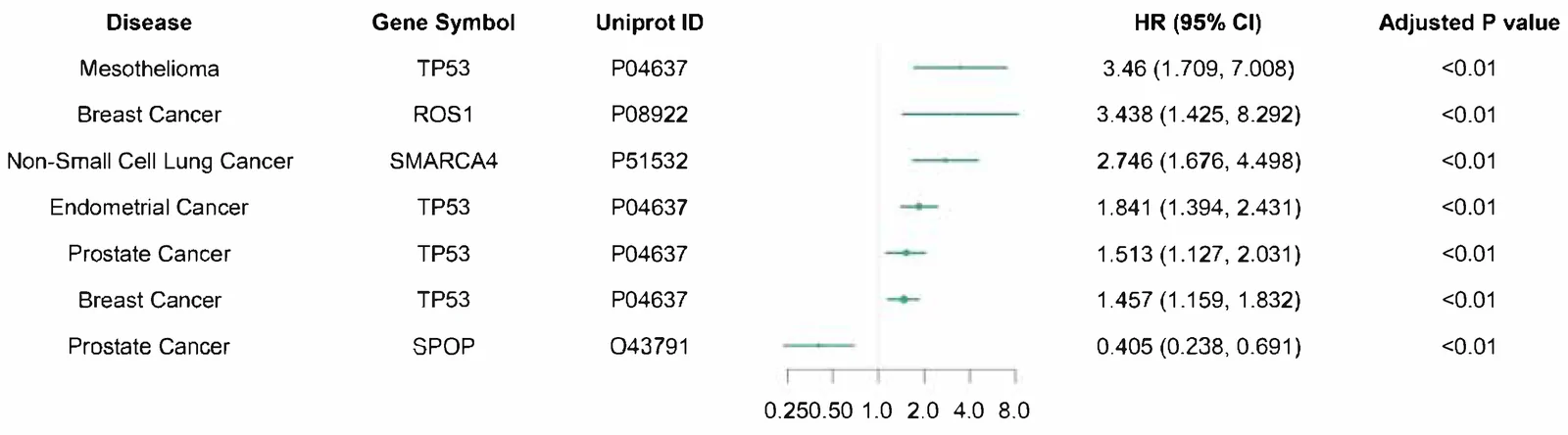
Mutations in early folding residues define a prognostically distinct subset across cancer types. Forest plot displaying 7 high-confidence associations between protein EFR mutations and patient survival outcomes in the MSK-MET cohort. Associations were identified using multivariate Cox proportional hazards regression. The columns list the cancer type (Disease), the target gene (Gene Symbol), the target protein (Uniprot ID), and the Hazard Ratio (HR) with 95% confidence intervals (95% CI). The green dots represent the estimated HR, while the horizontal lines denote the CI range. An HR > 1 indicates that mutations within the EFR of the specified protein are associated with increased mortality risk, whereas an HR < 1 indicates a protective effect. Adjusted *P*-values are provided for each association, highlighting significant prognostic markers (e.g., *TP53*/P04637 in multiple cancers). The adjusted *P* values shown are BH-adjusted *P* values from the multivariable Cox models.

To translate these findings into clinical utility, we performed a systematic survival screen to evaluate the prognostic potential of EFR-restricted mutations. We analyzed 570 protein–cancer combinations, specifically testing the survival outcomes of patients harboring mutations located within the predicted EFRs of a target protein. Initial screening identified 49 associations with nominal significance (*P* < 0.05; complete screening results are provided in Table S13 and Figures S4–S6). After applying a strict threshold (minimum five events per group and adjusted *P* < 0.05), 7 high-confidence pairs were retained for Cox proportional hazards analysis, revealing 6 risk-increasing and 1 risk-decreasing associations (Figure 5). Multivariate Cox proportional hazards analysis of these EFR-specific subsets revealed a predominant trend of poor prognosis, highlighting that distinct prognostic signals emerge only when mutational analysis is refined to focus specifically on early folding regions (Table S14).

**Figure 6:**
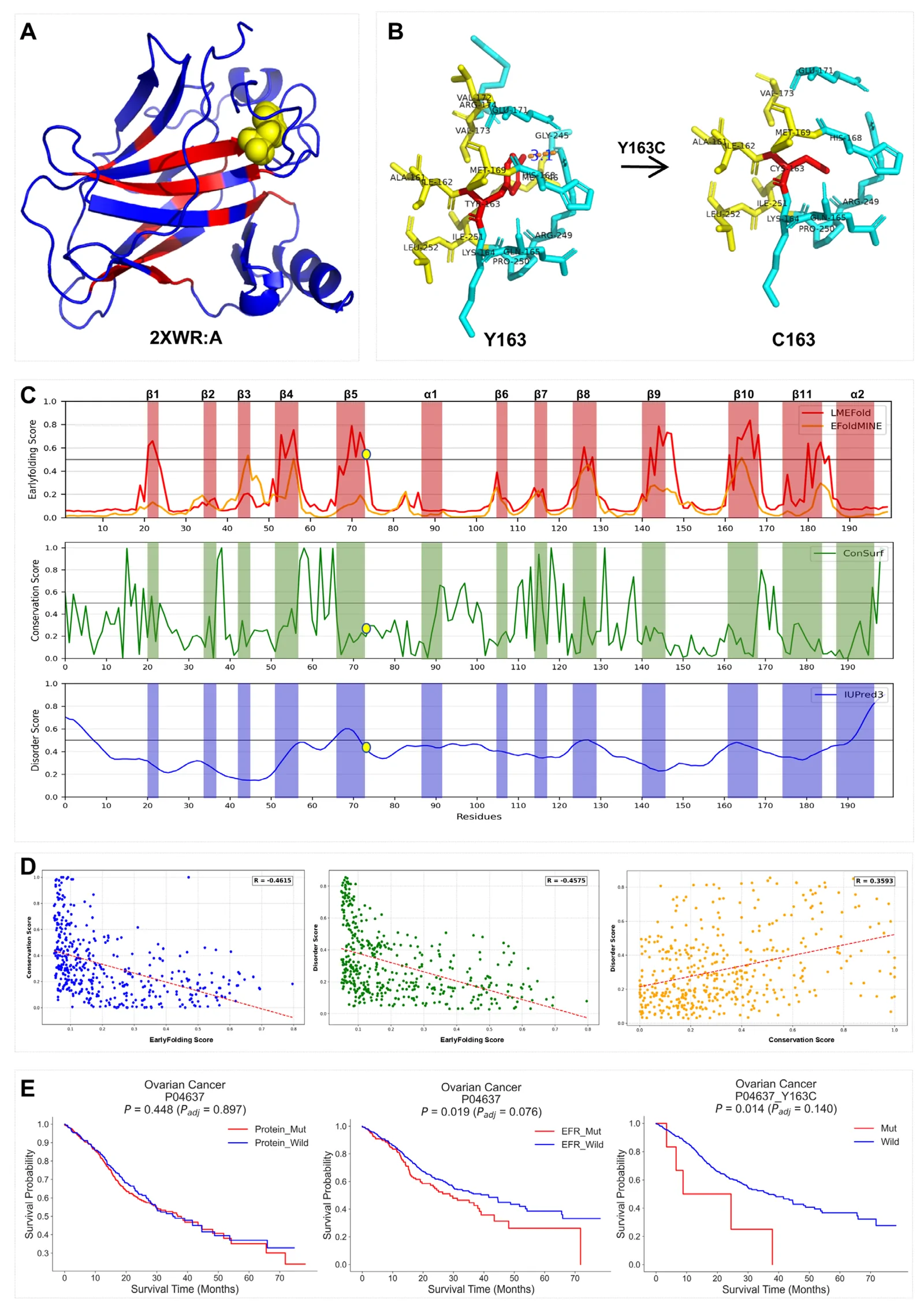
Disruption of early folding cores provides a mechanistic basis for pathogenicity and poor prognosis. (**A**) 3D structural mapping of predicted early folding signals on the *TP53* DNA-binding domain (PDB: 2XWR, chain A), where residue 1-199 directly correspond to the canonical positions 91-289 of human *TP53* (UniProt ID: P04637). Regions with high early folding propensity are colored red, and the hotspot residue Y163 is highlighted as yellow sphere. (**B**) Atomic modeling of the pathogenic p.Y163C mutation. Comparison between Wild-Type (Y163, left) and Mutant (C163, right) conformations reveals that the mutation introduces a shorter side chain, disrupting the hydrophobic packing network and increasing local atomic distances (red dashed lines), leading to structural destabilization. (**C**) Multi-dimensional sequence analysis. The tracks display LMEFold predicted scores (red), EFoldMine scores (orange), ConSurf conservation scores (green), and IUPred3 disorder scores (blue). The Y163 residue aligns perfectly with a peak in the early folding landscape (region β5) but shows intermediate conservation and low disorder. (**D**) Global correlation analysis. Scatter plots illustrate the relationships between residue-level early folding scores, evolutionary conservation, and intrinsic disorder across the protein sequence, revealing a negative correlation between early folding propensity and structural disorder. (**E**) Kaplan–Meier survival analysis in the MSK-MET ovarian cancer cohort. Overall *TP53* mutation status was not associated with survival (nominal *P* = 0.448; adjusted *P* = 0.897), whereas EFR-restricted mutations (nominal *P* = 0.019; adjusted *P* = 0.076) and *TP53* p.Y163C (nominal *P* = 0.014; adjusted *P* = 0.140) showed nominal associations with poorer survival. These associations did not remain significant after BH correction.

We next selected two paradigmatic tumor suppressors, *TP53* (UniProt: P04637) and *STK11* (UniProt: Q15831), as illustrative case studies. Specifically, we investigated the structural mechanism of the hotspot mutations *TP53* p.Y163C and *STK11* p.W308C. LMEFold correctly identified both Y163 and W308 as critical early folding sites (Figure 6C and Figure S7C). Structural modeling revealed that the Y163 side chain is deeply buried within the hydrophobic core of a central β-sheet cluster (Figure 6A). Its mutation to cysteine (Y163C) creates a significant packing void due to the substitution of the bulky tyrosine ring with a smaller sulfhydryl group. This “cavity-creating” mutation disrupts the native hydrophobic network and severs key hydrogen bonds, leading to severe local structural destabilization (Figure 6B), as quantitatively reflected by a substantially positive folding free energy change (ΔΔG = 3.14 ± 0.016 kcal/mol). Similarly, in LKB1 (encoded by *STK11*), the bulky hydrophobic side chain of W308 is critical for stabilizing the helical bundle; mutation to cysteine compromises local packing, inducing conformational rearrangement at the helix terminus (ΔΔG = 5.93 ± 0.034 kcal/mol, Figure S7A,B).

Intriguingly, multi-dimensional sequence profiling suggested that this vulnerability may stem from a fundamental evolutionary trade-off. As shown in Figure 6C, Y163 resides in a peak of early folding propensity (Region β5) but exhibits only intermediate conservation and low intrinsic disorder. Global correlation analysis confirmed this pattern: early folding propensity is negatively correlated with both sequence conservation (R ~ -0.46) and disorder (R ~ -0.46) (Figure 6D). This suggests that efficient early folding cores are not comprised of the most rigid or strongly conserved residues; rather, they reside within a metastable structural context that retains a degree of conformational plasticity to facilitate rapid folding. Consequently, mutations at these sites (like Y163C) are catastrophic because they disrupt this delicate stability-flexibility balance, exerting a disproportionately severe impact on the folding landscape compared to mutations at non-critical sites.

Most importantly, we illustrated the potential clinical relevance of this structural insight using a hierarchical stratification strategy of progressively increasing resolutions. In the MSK-MET ovarian cancer cohort, the global mutation status of *TP53* (Gene-level, Tier 1) failed to significantly stratify patient survival (*P* = 0.448). However, when we focused the analysis on EFR-resident mutations (EFR-level, Tier 2), a significant prognostic difference emerged (*P* = 0.019). Additionally, at the highest resolution (Site-level, Tier 3), the specific Y163C hotspot was strongly associated with poor survival (*P* = 0.014; Figure 6E). We observed a consistent trend for *STK11* in lung adenocarcinoma, where the specific W308C hotspot significantly stratified risk (*P* = 0.038; Figure S7E) while global mutation status did not. This stepwise improvement in *P*-values demonstrates that identifying mutations at early folding sites provides a potentially more granular prognostic stratification than traditional gene-level mutation profiling, resolving the “clinical noise” often seen in bulk mutational analyses. This hierarchical resolution demonstrates that EFR-based stratification provides a more precise framework for linking genetic variation to clinical outcomes than conventional gene-level analyses.

## 3. Discussion

Understanding how genetic variation disrupts protein folding remains a central challenge in molecular biology, yet the sequence-level determinants that govern folding initiation have remained largely unexplored. In this study, we identify early folding residues as a distinct class of structurally vulnerable sites that link protein sequence to mutational impact.

The prediction of EFRs has long been hindered by limited high-quality experimental data, creating a classic low-resource learning challenge. Here, we address this bottleneck by developing LMEFold, which transfers the “evolutionary wisdom” of large-scale PLMs to folding initiation prediction. Despite being trained on only 30 experimentally annotated proteins, LMEFold achieves a robust AUC of 0.831. This performance suggests that the self-attention mechanism of ESM-2 captures residue-level contextual dependencies that correlate with experimental folding signals. By bridging unsupervised pre-training and scarce experimental labels, LMEFold overcomes data limitations and achieves stable generalization across diverse protein families. In addition, the observed correlations between LMEFold scores and HDX-NMR protection factors in the PF-HDX-EvalSet provide complementary biophysical evidence. These protection factors reflect native-state protection rather than direct early-folding annotations.

With the widespread adoption of AlphaFold, a key question is whether static structural confidence alone can discriminate EFRs. To evaluate this, we compared LMEFold with the residue-level confidence metric pLDDT on 21 experimentally annotated proteins. LMEFold shows superior performance (AUC 0.829 vs. 0.765, Figure S2), indicating that sequence-derived features capture signals not fully reflected by structural confidence.

This observation reflects a fundamental difference in information representation between the two approaches: pLDDT quantifies the local confidence of static three-dimensional structure predictions, whereas LMEFold, trained on experimentally annotated data, learns statistical sequence features associated with early folding propensity. Therefore, in scenarios where reliable structural information is unavailable, where structural predictions exhibit limited confidence, or where static structures alone are insufficient to adequately characterize folding initiation processes, LMEFold can serve as a complementary approach, providing additional sequence-level information for identifying early folding sites.

The performance leap achieved by LMEFold over existing predictors highlights the necessity of capturing long-range dependencies in folding prediction. Previous approaches, largely relying on LSTM or CNN architectures, were constrained by local receptive fields, making them adept at identifying secondary structures but less effective at predicting the global hydrophobic collapse that drives early folding. By leveraging ESM-2 embeddings, LMEFold bypasses these local constraints. The Transformer’s self-attention mechanism effectively models the global context, allowing the network to capture how distant residues cooperate to stabilize the folding core. Our visualization of the latent feature space (Figure 2C) confirms this learning trajectory, showing that the model progressively disentangles the semantic representations of folding cores from the sequence background.

A key conceptual advance of this work is the proposal of the “evolutionary trade-off” hypothesis characterizing early folding sites. Our analysis of *TP53* and *STK11* reveals a paradox: while EFRs are physically essential for folding stability, they are not necessarily the most conserved or rigid residues. Instead, they appear to reside at delicate “evolutionary tipping points”—balancing the rigidity required to nucleate the core with the flexibility needed for functional dynamics. This dual requirement renders EFRs the critical determinants of structural vulnerability. Unlike active site mutations that may disable a specific function, mutations at EFRs (such as *TP53* p.Y163C and *STK11* p.W308C) disrupt the thermodynamic stability of the entire domain (ΔΔG > 1 kcal/mol), potentially triggering global misfolding or aggregation. This explains the specific enrichment of pathogenic variants in these regions (Figure 4) and supports the hypothesis that many “loss-of-function” diseases are fundamentally “loss-of-folding” pathologies.

Clinically, our results support a shift toward structure-aware precision medicine. Current oncological profiling often treats mutations within the same gene as equivalent (e.g., *TP53*-mutated vs. Wild-type). However, our survival analysis in the MSK-MET cohort demonstrates that this coarse classification masks significant prognostic heterogeneity. We showed that mutations specifically located within EFRs confer a significantly worse prognosis, whereas non-EFR mutations in the same gene may have a negligible impact on survival (Figure 6E). This distinction is critical for the interpretation of VUS. For VUS, LMEFold-derived EFR scores may help prioritize candidates for functional assays and further clinical investigation.

Despite these advances, our study has limitations. First, LMEFold is trained primarily on soluble globular proteins for which HDX data are available; its applicability to membrane proteins or intrinsically disordered proteins (IDPs), which follow distinct folding laws, remains to be fully validated. Second, our current model focuses on single-chain folding kinetics and does not yet account for co-translational folding or chaperone-mediated assembly in the crowded cellular environment. Although the Cox models were adjusted for age and sex, residual confounding cannot be excluded, particularly because treatment-related heterogeneity was not fully captured in the available cohort. Future iterations will aim to integrate multi-modal data, combining sequence embeddings with structural priors and protein–protein interaction networks, to decipher the complexity of multi-domain assembly. Nevertheless, by providing a robust computational microscope to view the earliest moments of protein life, LMEFold opens new avenues for understanding the molecular origins of disease and refining genotype–phenotype correlations in the era of genomic medicine.

## 4. Materials and Methods

### 4.1. Datasets and Cohorts

The training set of LMEFold (https://github.com/sduhoulab/EFR-pathogenic/; accessed on 1 October 2026) was constructed from high-quality experimental entries available in the public Start2Fold database (https://bio2byte.be/start2fold/; accessed on 1 October 2025) [32]. During data curation, only protein sequences with reliable residue-level experimental annotations and pulse-labeling or equivalent HDX information were retained, and sequence length was constrained to minimize potential confounding effects of extreme length on folding kinetics analysis. The resulting dataset consists of 30 protein sequences, covering 3,398 experimentally supported residues, among which 482 residues are explicitly annotated as early-folding sites in Start2Fold (14.18%). This dataset, referred to as Benchmark30 (Table S1), is identical to the original training set used by EFoldMine, (https://bio2byte.be/b2btools/efoldmine/; accessed on 1 October 2025), enabling direct and standardized performance comparison across different methods.

To evaluate the predictive performance and generalizability of LMEFold, we employed two distinct validation approaches. Ubiquitin (PDB: 1UBQ), a structurally compact protein excluded from training, was used as an independent case to qualitatively examine whether LMEFold captures sequence-encoded early folding features (Table S2). Furthermore, we incorporated the PF-HDX-EvalSet (https://doi.org/10.1016/j.str.2021.10.006; accessed on 1 October 2025) as a quantitative benchmark (Table S3), which consists of 431 residues from 10 proteins with experimentally determined protection factors (PFs) measured via HDX-NMR [23]. The PF is defined as the ratio of the intrinsic hydrogen exchange rate to the experimentally measured rate, where higher log(PF) values correspond to greater structural protection, thereby providing an experimental baseline to validate the correlation between LMEFold scores and biophysical parameters.

This study utilizes de-identified public data in accordance with relevant ethical guidelines and human subject protection requirements. The pathogenic and clinical significance of mutations occurring at EFRs was investigated by integrating multiple sources of germline and somatic variant data. Germline variants were collected from ClinVar (https://www.ncbi.nlm.nih.gov/clinvar/; October 2024 release; accessed on 1 October 2025), gnomAD (v2.1.1) [24,25], and the UK Biobank (UKB; https://www.ukbiobank.ac.uk/; accessed on 1 October 2025) [26,27]. ClinVar aggregates human genetic variants and their clinical significance submitted by both clinical and research communities, serving as an important public repository for disease-associated mutations [24]. gnomAD consolidates large-scale exome and genome sequencing efforts, systematically summarizing variant frequencies and selection constraints in the general population [25]. The UKB provides a high-quality resource of genotype and phenotype data encompassing both common and rare variants [26,27]. This research has been conducted using the UK Biobank Resource under Application Number 98273. Together, gnomAD and UKB establish the baseline genetic background of the general population, providing reference controls for comparison with disease-associated mutations. Following unified quality control and filtering, missense variants were extracted from each database and deduplicated. In total, we retained 23,369 pathogenic missense variants from ClinVar (filtered from 29,187 entries); 1,952,086 variants from UKB (from 1,954,521 entries); and 4,667,175 variants from gnomAD (from 4,672,347 entries).

Somatic variant data were obtained from the MSK-MET cohort, (available through cBioPortal: https://www.cbioportal.org/study/summary?id=msk_met_2021; accessed on 1 October 2025), which encompasses pan-cancer mutational profiles and follow-up clinical information [28]. From this dataset, we extracted 129,411 somatic nonsynonymous single-nucleotide variants (nsSNVs) along with corresponding clinical records, enabling a systematic investigation of the relationship between EFR mutations and patient prognosis.

### 4.2. Feature Extraction and Visualization

To obtain informative representations for each residue, we utilized pre-trained PLMs to convert amino acid sequences into high-dimensional latent embeddings. We systematically evaluated four representative architectures: ProtBERT, ProtT5, and two variants of the ESM-2 family, specifically the 35M-parameter model (ESM-2-t12) and the 150M-parameter model (ESM-2-t30). These models, pre-trained on massive sequence databases, capture implicit evolutionary and physicochemical constraints governing protein structure.

For a given input sequence, the corresponding tokenizer first segments the amino acids, which are then processed by the model backbone. ProtBERT and ProtT5 yield 1024-dimensional embeddings per residue, while the ESM-2-t30 model generates 640-dimensional embeddings. These vectors were used as the primary input features for the downstream classification task. By relying on pre-trained embeddings, the model can leverage patterns learned from millions of protein sequences, which is particularly beneficial for tasks where experimental annotations are limited.

To further interpret the learning mechanism as described in the interpretability study, we employed the t-distributed Stochastic Neighbor Embedding (t-SNE) algorithm. Latent representations were extracted from intermediate and final network layers during the training process and projected into a two-dimensional space, allowing us to trace the progressive separation of early-folding and non-early-folding residue clusters.

### 4.3. Model Training

To map the residue-level latent representations extracted by the pre-trained PLMs into binary predictions, we adopted a token classification architecture. Specifically, a dropout layer was applied to mitigate overfitting. This was followed by a single linear projection layer that mapped the high-dimensional hidden representations directly into two logits, corresponding to the EFR and non-EFR classes. The input dimension of the projection layer was 1024 for ProtBERT and ProtT5, 480 for ESM-2-t12, and 640 for ESM-2-t30. The dropout rate was selected during hyperparameter optimization.

Given the limited size of the Benchmark30 dataset (*n* = 30) and the potential for sequence homology, we implemented a rigorous homology-aware nested cross-validation framework to prevent information leakage and enable a reliable assessment of model generalization. All protein sequences were sequentially clustered using the CD-HIT suite v4.8.1 [33]. CD-HIT was applied at 60% sequence identity (-c 0.60, -n 4), followed by PSI-CD-HIT v4.8.1 clustering at 30% and 25% identity, yielding 27 mutually exclusive sequence clusters. This procedure ensured that homologous proteins were assigned to the same cluster and prevented closely related sequences from appearing simultaneously in training and testing sets.

Subsequently, a group-wise LOOCV scheme was employed in the outer loop. In each iteration, one cluster was held out as an independent test set, while the remaining 26 clusters were combined to form the training candidate set. Within the inner loop, the training candidate set was subjected to 5-fold cross-validation for hyperparameter optimization. The model was then retrained using all 26 training clusters with the selected hyperparameters and evaluated on the outer-loop held-out test cluster. This process was repeated 27 times, such that each cluster served exactly once as an independent test set. Throughout this procedure, hyperparameter optimization was performed exclusively within the inner-loop training data, while the outer-loop test proteins remained completely unseen until final evaluation. Final model performance was reported as the mean ± standard deviation of the AUC across the 27 outer-loop evaluations.

To address the pronounced class imbalance arising from the low proportion of early-folding residues, a class-weighted cross-entropy loss was employed during model training.

(1) $$\mathrm{Weighted}\;\mathrm{Cross}\;\mathrm{Entropy}\;\mathrm{Loss}(x,y)=-\;\frac{1}{N}\sum_{i=1}^{N}w_{y_{i}}.\log(p_{y_{i}})$$

Here, *N* denotes the number of samples; *y_i_* ∈ {0,1} indicates the true label of the *i*-th sample (1 denotes EFR, 0 denotes non-EFR); *p_i_*∈ [0,1] represents the predicted probability of the *i*-th sample being an EFR; and $w_{y_{i}}$ denotes the class weight. To increase the contribution of minority-class samples during optimization, weighted loss functions were adopted. To assess the robustness of the weighting strategy, sensitivity analyses were performed using multiple positive-class weights, and the detailed results are provided in Table S6.

To ensure efficient convergence and reduce the risk of overfitting in this low-resource setting, model training incorporated multiple optimization and regularization strategies, including the BertAdam optimizer, independent validation-based model selection, learning-rate scheduling, gradient clipping, and early stopping. To guarantee reproducibility, the random seed was fixed at 42 and the maximum number of training epochs was set to 20. Validation AUC was used as the monitoring metric, and training was terminated when no improvement greater than 0.001 was observed for five consecutive epochs. The model achieving the highest validation AUC was retained as the final model.

### 4.4. Computation of Evolutionary and Biophysical Descriptors

To characterize the biological properties of EFRs, we integrated multiple evolutionary and conformational descriptors. Residue-level evolutionary conservation was quantified using the normalized evolutionary-rate scores reported in the ‘SCORE’ column of the ConSurf output [34,35]. These scores were normalized by ConSurf to a mean of zero and a standard deviation of one, with lower values indicating greater conservation. The intrinsic disorder propensity of each residue was quantified using the IUPred3 (https://iupred3.elte.hu/; accessed on 1 October 2025) [36] algorithm. Furthermore, to evaluate the functional consequences of mutations, we retrieved SIFT [29] and PolyPhen-2 [30] scores through the Ensembl Variant Effect Predictor (VEP version 111) [31]. These scores provided a standardized baseline to compare the predicted deleteriousness of substitutions occurring at early folding sites versus those at other positions. The cumulative distributions of these scores were compared between groups to assess whether EFRs exhibit a higher sensitivity to mutational perturbations.

### 4.5. Evaluation Metrics

The performance of LMEFold was evaluated using a suite of standard classification metrics including AUC, accuracy, F1 score, recall, precision, and specificity. Given the class imbalance in the Benchmark30 dataset, where EFRs represent 14.18% of the total, the AUC was selected as the primary metric to ensure a threshold-independent assessment of discriminative power. These metrics were calculated based on the counts of True Positives (TP), True Negatives (TN), False Positives (FP), and False Negatives (FN), as defined in Equations (2)–(7). Specifically, TP and TN represent correctly predicted EFRs and non-EFRs, respectively, while FP and FN denote the corresponding misclassifications:

AUC − ROC: area under the cuve of the ROC plot(2)

Accuracy = (TP + TN)/(TP + FN + TN + FP)(3)

(4) F1=2×Precision×Recall/(Precision+ Recall)

Recall (True Pos. Rate, TPR, Sensitivity, Coverage) = TP/(TP + FN)(5)

(6) Precision (Positive Predictive Value, PPV)=TP/(TP+FP)

Specificity (True Negative Rate, TNR) = TN/(TN + FP) (7)

Additionally, for the quantitative validation on the PF-HDX-EvalSet, we utilized the residue-level Pearson correlation coefficient (r) to quantify the linear relationship between the model-predicted probability scores and the experimental log(PF) values.

### 4.6. Functional and Clinical Analysis of Mutations at Early Folding Sites

We integrated the LMEFold predictions with multiple variant databases and clinical cohorts. Missense variants were collected from ClinVar, UKB, and gnomAD. ClinVar provided pathogenic variants, retaining only those with a review status of ≥1 star and annotated as Pathogenic, Likely Pathogenic, or Pathogenic/Likely Pathogenic, forming the “pathogenic mutation group”. Variants from UKB and gnomAD, representing population backgrounds, were filtered for high-quality (“PASS”) common missense variants to construct the “control mutation group”. All variants were precisely mapped to protein residues using the VEP to ensure coordinate consistency between genomic loci and residue positions, and the LMEFold predictions were used to determine whether each variant was located within an EFR. For analyses requiring binary EFR labels, residues with LMEFold probability scores greater than or equal to 0.58 were classified as EFRs. This cutoff was selected by maximizing the F1 score exclusively on the validation set and was then fixed for the held-out test set and the subsequent ClinVar, UK Biobank, gnomAD, and MSK-MET analyses. The proportion of EFRs-associated variants was then calculated for each dataset. By comparing the distribution of EFR variants between the two groups, we evaluated whether EFRs represent regions enriched for pathogenic mutations. Statistical significance of distributional differences was assessed using the Mann–Whitney U test, thereby revealing potential functional effects of EFR mutations.

Subsequently, we investigated the association between EFR mutations and patient survival using the MSK-MET pan-cancer genomic–clinical cohort, aiming to extend the preceding finding that “EFRs represent regions enriched for pathogenic mutations” to a clinical prognostic context. Specifically, somatic nsSNVs and corresponding follow-up data were systematically extracted from the cohort and precisely mapped to the protein level using UniProt’s ID Mapping tool (https://www.uniprot.org/id-mapping; accessed on 1 October 2025). The LMEFold model was then applied to determine whether each mutation was located within an EFR, ensuring analytical accuracy.

We implemented a hierarchical stratification strategy to isolate the prognostic impact of EFR-specific perturbations from overall gene mutational status. At the first tier, we evaluated the baseline prognostic effect of any missense mutation within a given protein compared to the wild-type state. At the second tier, we compared the survival outcomes of patients harboring mutations specifically within predicted EFRs against a combined control group consisting of patients with mutations in non-EFR regions or no mutations in the corresponding proteins. Furthermore, we conducted a site-specific analysis focusing on high-frequency pathogenic hotspots such as *TP53* p.Y163C to validate the impact of individual folding-critical residues. To minimize bias from small sample sizes and ensure robustness, cancer type–mutation combinations with sample sizes <5 were excluded. For the remaining combinations, survival outcomes were assessed using Kaplan–Meier estimates with Log-Rank tests (detailed results are provided in Figures S4–S6 and Table S13). The log-rank *P* values were adjusted using the Benjamini–Hochberg procedure, and associations with adjusted *P* < 0.05 were entered into multivariable Cox proportional hazards regression adjusted for age and sex. The Cox-model *P* values were subsequently adjusted using the Benjamini–Hochberg procedure, with adjusted *P* < 0.05 defining the final significant associations.

### 4.7. Structural Modeling and Energetics Calculation

To elucidate the molecular basis of mutational disruption, we performed structural analysis and thermodynamic stability calculations. The three-dimensional structures of TP53 (PDB: 2XWR) and STK11 (AlphaFold prediction: AF-Q15831-F1-model_v4.pdb) served as templates for mutational modeling, with coordinate mappings between PDB structures and canonical UniProt sequences provided in Table S15. We utilized FoldX version 5.0 to calculate the change in folding free energy (ΔΔG) resulting from specific amino acid substitutions. The structural models were first processed using the RepairPDB command, and the BuildModel command was executed with five independent runs to ensure robustness. For visualization, residue-level prediction scores were encoded into the B-factor column of the PDB files. This allowed for the spatial visualization of folding initiation propensities on the three-dimensional structures, including myoglobin (PDB: 1MBC) and ubiquitin (PDB: 1UBQ), using PyMOL version 2.5 to analyze hydrophobic packing and hydrogen bonding networks.

## 5. Conclusions

In this study, we address the long-standing challenge of predicting EFRs under limited experimental data by leveraging PLMs to extract folding-relevant signals directly from sequence. Beyond predictive performance, we propose an exploratory hypothesis: EFRs are shaped by an evolutionary trade-off between structural stability and functional flexibility, positioning them as critical yet intrinsically vulnerable elements of protein architecture. Mutations at these sites preferentially disrupt folding thermodynamics rather than local function, providing a mechanistic basis for their enrichment in disease and their strong prognostic impact. Together, these findings shift the interpretation of genetic variation from a static, gene-centric view toward a folding-centric, structure-resolved perspective, providing a framework for interpreting mutation effects.

## Figures and Tables

**Table 1:** Hyperparameter categories and search ranges.

Hyperparameter	Training Significance	Candidate Values
learning rate	Controls the step size of parameter updates	1 × 10^−5^, 2 × 10^−5^, 5 × 10^−5^
dropout rate	Randomly discards neuron connections to mitigate overfitting	0.01, 0.05, 0.1, 0.2
batch size	Affects the stability of gradient estimation and training efficiency	1, 2, 4

**Table 2:** Comparison of LMEFold with other models.

	AUC	Acc	F1	Recall	Pre	Spe
ProtBERT	0.800 ± 0.102	0.773 ± 0.113	0.385 ± 0.141	0.540 ± 0.259	0.371 ± 0.203	0.814 ± 0.150
ProtT5	0.804 ± 0.088	0.783 ± 0.122	0.380 ± 0.142	0.508 ± 0.234	0.378 ± 0.198	0.842 ± 0.147
ESM-2-t12	0.812 ± 0.073	0.759 ± 0.076	0.389 ± 0.150	0.604 ± 0.220	0.349 ± 0.202	0.798 ± 0.111
EFoldMine	0.805 ± 0.081	0.727 ± 0.086	**0.414** **±** **0.157**	**0.731** **±** **0.179**	0.336 ± 0.202	0.739 ± 0.119
**LMEFold**	**0.831** **±** **0.068**	**0.807** ± **0.051**	0.390 ± 0.123	0.513 ± 0.244	**0.403** **±** **0.207**	**0.875** **±** **0.070**

Bold values indicate the best performance for each metric.

## Data Availability

The data analyzed in this study were obtained from the following publicly available and controlled-access resources: Start2Fold (https://bio2byte.be/start2fold/; accessed on 1 October 2025; DOI: 10.1093/nar/gkv1185) [32], PF-HDX-EvalSet (https://doi.org/10.1016/j.str.2021.10.006) [23], RCSB Protein Data Bank (https://www.rcsb.org/; PDB IDs: 1MBC, 1UBQ, and 2XWR; accessed on 1 October 2025), AlphaFold Protein Structure Database (https://alphafold.ebi.ac.uk/; UniProt accession: Q15831; accessed on 1 October 2025), ClinVar (https://www.ncbi.nlm.nih.gov/clinvar/; accessed on 1 October 2025) [24], gnomAD (https://gnomad.broadinstitute.org/; accessed on 1 October 2025) [25], UK Biobank (https://www.ukbiobank.ac.uk/; Application Number 98273; accessed on 1 October 2025) [26,27], and the MSK-MET pan-cancer cohort via cBioPortal (study ID: msk_met_2021; https://www.cbioportal.org/study/summary?id=msk_met_2021; accessed on 1 October 2025; Zenodo DOI: 10.5281/zenodo.5801902) [28]. Access to controlled-access datasets, including UK Biobank data, is subject to the applicable policies and permissions of the respective data providers. The source code for the LMEFold framework, including data preprocessing, model training, and inference scripts, is available at https://github.com/sduhoulab/EFR-pathogenic/ (accessed on 1 October 2026).The residue-level prediction outputs used to generate the results in Table 2, including those for LMEFold (using ESM-2-t30 as the backbone), EFoldMine, ProtBERT, ProtT5, and ESM-2-t12, are publicly available at https://github.com/sduhoulab/EFR-pathogenic/tree/main/data (accessed on 1 October 2026). The 27 trained fold-specific LMEFold models are publicly available at https://huggingface.co/LMEFold/models (accessed on 1 October 2026), and an online prediction interface integrating these models is available at https://huggingface.co/spaces/sduhoulab/LMEFold_Agent (accessed on 1 October 2026). The processed data supporting the findings of this study are provided within the article and its Supplementary Materials.

## Supplementary Materials

The following supporting information can be downloaded at: https://www.mdpi.com/article/10.3390/ijms27198914/s1.

## References

[1] Backwell L., Marsh J.A. (2022). Diverse Molecular Mechanisms Underlying Pathogenic Protein Mutations: Beyond the Loss-of-Function Paradigm. Annu. Rev. Genom. Hum. Genet..

[2] Sahni N., Yi S., Taipale M., Bass J.I.F., Coulombe-Huntington J., Yang F., Peng J., Weile J., Karras G.I., Wang Y. (2015). Widespread Macromolecular Interaction Perturbations in Human Genetic Disorders. Cell.

[3] Stein A., Fowler D.M., Hartmann-Petersen R., Lindorff-Larsen K. (2019). Biophysical and Mechanistic models for disease-causing protein variants. Trends Biochem. Sci..

[4] Cheng J., Novati G., Pan J., Bycroft C., Žemgulytė A., Applebaum T., Pritzel A., Wong L.H., Zielinski M., Sargeant T. (2023). Accurate proteome-wide missense variant effect prediction with AlphaMissense. Science.

[5] Starita L.M., Ahituv N., Dunham M.J., Kitzman J.O., Roth F.P., Seelig G., Shendure J., Fowler D.M. (2017). Variant Interpretation: Functional Assays to the Rescue. Am. J. Hum. Genet..

[6] Krokidis M.G., Dimitrakopoulos G.N., Vrahatis A.G., Exarchos T.P., Vlamos P. (2024). Challenges and limitations in computational prediction of protein misfolding in neurodegenerative diseases. Front. Comput. Neurosci..

[7] Chiti F., Dobson C.M. (2017). Protein Misfolding, Amyloid Formation, and Human Disease: A Summary of Progress Over the Last Decade. Annu. Rev. Biochem..

[8] Flagg M.P., Lam B., Lam D.K., Le T.M., Kao A., Slaiwa Y.I., Hampton R.Y. (2023). Exploring the “misfolding problem” by systematic discovery and analysis of functional-but-degraded proteins. Mol. Biol. Cell.

[9] Daggett V., Fersht A.R. (2003). Is there a unifying mechanism for protein folding?. Trends Biochem. Sci..

[10] Li A., Daggett V. (1998). Molecular dynamics simulation of the unfolding of barnase: Characterization of the major intermediate1. J. Mol. Biol..

[11] Bhutani N., Udgaonkar J.B. (2003). Folding subdomains of thioredoxin characterized by native-state hydrogen exchange. Protein Sci..

[12] Dobson C.M. (2003). Protein folding and misfolding. Nature.

[13] James E.I., Murphree T.A., Vorauer C., Engen J.R., Guttman M. (2022). Advances in Hydrogen/Deuterium Exchange Mass Spectrometry and the Pursuit of Challenging Biological Systems. Chem. Rev..

[14] Dyson H.J., Wright P.E. (2004). Unfolded Proteins and Protein Folding Studied by NMR. Chem. Rev..

[15] Masson G.R., Burke J.E., Ahn N.G., Anand G.S., Borchers C., Brier S., Bou-Assaf G.M., Engen J.R., Englander S.W., Faber J. (2019). Recommendations for performing, interpreting and reporting hydrogen deuterium exchange mass spectrometry (HDX-MS) experiments. Nat. Methods.

[16] Raimondi D., Orlando G., Pancsa R., Khan T., Vranken W.F. (2017). Exploring the Sequence-based Prediction of Folding Initiation Sites in Proteins. Sci. Rep..

[17] Grau I., Nowé A., Vranken W. (2021). Interpreting a black box predictor to gain insights into early folding mechanisms. Comput. Struct. Biotechnol. J..

[18] Jumper J., Evans R., Pritzel A., Green T., Figurnov M., Ronneberger O., Tunyasuvunakool K., Bates R., Žídek A., Potapenko A. (2021). Highly accurate protein structure prediction with AlphaFold. Nature.

[19] Rives A., Meier J., Sercu T., Goyal S., Lin Z., Liu J., Guo D., Ott M., Zitnick C.L., Ma J. (2021). Biological structure and function emerge from scaling unsupervised learning to 250 million protein sequences. Proc. Natl. Acad. Sci. USA.

[20] Elnaggar A., Heinzinger M., Dallago C., Rehawi G., Wang Y., Jones L., Gibbs T., Feher T., Angerer C., Steinegger M. (2022). ProtTrans: Toward Understanding the Language of Life Through Self-Supervised Learning. IEEE Trans. Pattern Anal. Mach. Intell..

[21] Lin Z., Akin H., Rao R., Hie B., Zhu Z., Lu W., Smetanin N., Verkuil R., Kabeli O., Shmueli Y. (2023). Evolutionary-scale prediction of atomic-level protein structure with a language model. Science.

[22] Meier J., Rao R., Verkuil R., Liu J., Sercu T., Rives A. (2021). Language models enable zero-shot prediction of the effects of mutations on protein function. Adv. Neural Inf. Process. Syst..

[23] Nguyen T.T., Marzolf D.R., Seffernick J.T., Heinze S., Lindert S. (2022). Protein structure prediction using residue-resolved protection factors from hydrogen-deuterium exchange NMR. Structure.

[24] Landrum M.J., Chitipiralla S., Kaur K., Brown G., Chen C., Hart J., Hoffman D., Jang W., Liu C., Maddipatla Z. (2024). ClinVar: Updates to support classifications of both germline and somatic variants. Nucleic Acids Res..

[25] Karczewski K.J., Francioli L.C., Tiao G., Cummings B.B., Alfoldi J., Wang Q., Collins R.L., Laricchia K.M., Ganna A., Birnbaum D.P. (2020). The mutational constraint spectrum quantified from variation in 141,456 humans. Nature.

[26] Sudlow C., Gallacher J., Allen N., Beral V., Burton P., Danesh J., Downey P., Elliott P., Green J., Landray M. (2015). UK Biobank: An Open Access Resource for Identifying the Causes of a Wide Range of Complex Diseases of Middle and Old Age. PLoS Med..

[27] Van Hout C.V., Tachmazidou I., Backman J.D., Hoffman J.D., Liu D., Pandey A.K., Gonzaga-Jauregui C., Khalid S., Ye B., Banerjee N. (2020). Exome sequencing and characterization of 49,960 individuals in the UK Biobank. Nature.

[28] Nguyen B., Fong C., Luthra A., Smith S.A., DiNatale R.G., Nandakumar S., Walch H., Chatila W.K., Madupuri R., Kundra R. (2022). Genomic characterization of metastatic patterns from prospective clinical sequencing of 25,000 patients. Cell.

[29] Ng P.C., Henikoff S. (2003). SIFT: Predicting amino acid changes that affect protein function. Nucleic Acids Res..

[30] Adzhubei I.A., Schmidt S., Peshkin L., E Ramensky V., Gerasimova A., Bork P., Kondrashov A.S., Sunyaev S.R. (2010). A method and server for predicting damaging missense mutations. Nat. Methods.

[31] McLaren W., Gil L., Hunt S.E., Riat H.S., Ritchie G.R.S., Thormann A., Flicek P., Cunningham F. (2016). The Ensembl Variant Effect Predictor. Genome Biol..

[32] Pancsa R., Varadi M., Tompa P., Vranken W.F. (2016). Start2Fold: A database of hydrogen/deuterium exchange data on protein folding and stability. Nucleic Acids Res..

[33] Li W., Godzik A. (2006). Cd-hit: A fast program for clustering and comparing large sets of protein or nucleotide sequences. Bioinformatics.

[34] Glaser F., Pupko T., Paz I., Bell R.E., Bechor-Shental D., Martz E., Ben-Tal N. (2003). ConSurf: Identification of Functional Regions in Proteins by Surface-Mapping of Phylogenetic Information. Bioinformatics.

[35] Ashkenazy H., Abadi S., Martz E., Chay O., Mayrose I., Pupko T., Ben-Tal N. (2016). ConSurf 2016: An improved methodology to estimate and visualize evolutionary conservation in macromolecules. Nucleic Acids Res..

[36] Erdős G., Pajkos M., Dosztányi Z. (2021). IUPred3: Prediction of protein disorder enhanced with unambiguous experimental annotation and visualization of evolutionary conservation. Nucleic Acids Res..

