# Supplementary material for: Protein Language Models Identify Early Folding Residues as Critical Hotspots for Pathogenic Variants and Cancer Prognosis
Source: Int J Mol Sci. 2026 Oct 7;27(19):8914. doi: 10.3390/ijms27198914 (PMC13666245; doi:10.3390/ijms27198914)
Supplement: Supplementary file 1 [file ijms-27-08914-s001.zip › ijms-4572590-supplementary.pdf]

SUPPLEMENTARY DATA

Supplementary section S1: Benchmark30 set

The training set of LMEFold was derived from high-quality experimental entries in the publicly available Start2Fold database and comprises 30 curated protein sequences.

Table S1 Start2Fold High-Quality Early Folding Residue Dataset

Length	ID	Sequence
56	1omu	LAAVSVDCSEYPKPACTLEYRPLCGSDNKTYGNKCNFCNAVVESNGTLTLSHFGK<br>C
56	1pga	MTYKLILNGKTLKGETTTEAVDAATAEKVFKQYANDNGVDGEWTYDDATKTFTV<br>TE
58	5pti	RPDFCLEPPYTGPCKARIIRYFYNAKAGLCQTFVYGGCRAKRNNFKSAEDCMRTCG<br>GA
60	1bdd	TADNKFNKEQQNAFYEILHLPNLNEEQRNGFIQSLKDDPSQSANLLAEAKKLNDAQ<br>APKA
60	2crt	LKCNKLVPLFYKTCPAGKNLCYKMFMVATPKVPVKRGCIDVCPKSSLLVKYVCCN<br>TDRCN
62	1coe	LECHNQQSSQTPTTTGCSGGETNCYKKRWRDHRGYRTERGCGCPSVKNGIEINCCT<br>TDRCNN
78	2ptl	ENKEETPETPETDSEEEVTIKANLIFANGSTQTAEFKGTFEKATSEAYAYADTLKKD<br>NGEYTVDVADKGYTLNIKFAG
86	2abd	SQAEFDKAAEEVKHLKTKPADEEMLFIYSHYKQATVGDINTERPGMLDFKGKAKW<br>DAWNELKGTSKEDAMKAYIDKVEELKKKYGI
97	1a64	RDSGTVWGALGHGINLNIPNFQMTDDIDEVRWERGSTLVAEFKRKPFLKSGAFEIL<br>ANGDLKIKNLTRDDSGTYNVTVYSTNGTRILDKALDLRILE
99	9pcy	LEVLLGSGDGSLVFVPSEFSVPSGEKIVFKNNAGFPHNVVFDEDEIPAGVDAVKISM<br>PEEELLNAPGETYVVTLDTKGTYSFYCSPHQGAGMVGKVTVN
104	1onc	EDWLTFQKKHITNTRDVDCDNIMSTNLFHCKDKNTFIYSRPEPVKAICKGIIASKNV<br>LTTSEFYLSDCNVTSRPCKYKLKKSTNKFCVTCENQAPVHFVGVGSC
104	1hrc	GDVEKGKKIFVQKCAQCHTVEKGGKHKTGPNLNGLFGRKTGQAPGFTYTDANKN<br>KGITWKEETLMEYLENPKKYIPGTKMIFAGIKKKTEREDLIAYLKKATNE
104	1e3y	GSHMGEEDLCAAFNVICDNVGKDWRRLARQLKVSDTKIDSIEDRYPRNLTERVRES<br>LRIWKNTEKENATVAHLVGALRSCQMNLVADLVQEVQQARDLQNRSGA
104	1ygw	ACDYTCGSNCYSSSDVSTAQAAGYKLHEDGETVGSNSYPHKYNNYEGFDFSVSSP<br>YYEWPILSSGDVYSGGSPGADRVVFNENNQLAGVITHTGASGNNFVECT
118	1hce	MGNRAFKSHHGHFLSAEGEAVKTHHGHHDHHTHFHVENHGGKVALKTHCGKYL<br>SIGDHKQVYLSHHLHGDHSLFHLEHHGGKVSIKGHHHHYISADHHGHVSTKEHHD<br>HDTTFEEIII
124	1rbx	KETAAAKFERQHMDSSTSAASSSNYCNQMMKSRNLTKDRCKPVNTFVHESLADV<br>QAVCSQKNVACKNGQTNCYQSYSTMSITDCRETGSSKYPNCAYKTTQANKHIIVA<br>CEGNPYVPVHFDASV
126	2vil	VELSKKVTGKLDKTTPGIQIWRIENMEMVPVPTKSYGNFYEGDCYVLLSTRKTGSG<br>FSYNIHYWLGKNSSQDEQGAAAIYTTQMDEYLGSVAVQHREVQGHESETFRAYFK

		QGLIYKQGGVASGMK
129	1hel	KVFGRCELAAAMKRHGLDNYRGYSLGNWVCAAKFESNFNTQATNRNTDGSTDY<br>GILQINSRWWCNDGRTPGSRNLCNIPCSALLSSDITASVNCAKKIVSDGNGMNAWV<br>AWRNRCKGTDVQAWIRGCRL
129	2eql	KVFSKCELAHKLKAQEMDGFGGYSLANWVCMAEYESNFNTRAFNGKNANGSSDY<br>GLFQLNNKWWCKDNKRSSSNACNIMCSKLLDENIDDDISCAKRVVRDPKGMSAW<br>KAWVKHCKDKDLSEYLASCNL
130	1lz1	KVFERCELARTLKRLGMDGYRGISLANWMCLAKWESGYNTRATNYNAGDRSTDY<br>GIFQINSRYWCNDGKTPGAVNACHLSCSALLQDNIADAVACAKRVVRDPQGIRAW<br>VAWRNRCQNRDVRQYVQGCGV
130	1hrh	YQLEKEPIVGAETFYVDGAANRETKLGKAGYVTNKGRQKVVPLTNTTNQKTELQA<br>IYLALQDSGLEVNIVTDSQYALGIIQAQPDKSESELVNQIIEQLIKKEKVYLAWVPXX<br>XXXGGNEQVDKLVSAGI
140	1rg8	FNLPPGNYKKPKLLYCSNGGHFLRILPDGTVDGTRDRSDQHIQLQLSAESVGEVYIK<br>STETGQYLAMDTDGLLYGSQTPNEECLFLERLEENHYNTYISKKHAEKNWFVGLK<br>KNGSCKRGPRTHYGQKAILFLPLPVSSD
149	1joo	ATSTKKLHKEPATLIKAIDGDTVKLMYKGQPMTFRLLLVDTPETKHPKKGVEKYGP<br>EASAFTKKMVENAKKIEVEFDKGQRTDKYGRGLAYIYADGKMVNEALVRQGLAK<br>VAYVYKPNNTHEQLLRKSEAQAKKEKLNIWSEDNADSGQ
153	1ymb	GLSDGEWQQVLNVWGKVEADIAGHGQEVLIRLFTGHPETLEKFDKFKHLKTEAEM<br>KASEDLKKHGTVVLTALGGILKKKGHHEAELKPLAQSHATKHKIPIKYLEFISDAIIH<br>VLHSKHPGDFGADAQGAMTKALELFRNDIAAKYKELGFQG
153	1mbc	VLSEGEWQLVLHVWAKVEADVAGHGQDILIRLFKSHPETLEKFDRFKHLKTEAEM<br>KASEDLKKHGVTVLTALGAILKKKGHHEAELKPLAQSHATKHKIPIKYLEFISEAIIH<br>VLHSRHPGDFGADAQGAMNKALELFRKDIAAKYKELGYQG
153	1i1b	APVRSLNCTLRDSQQKSLVMSGPYELKALHLQGQDMEQQVVFSMSFVQGEESNDK<br>IPVALGLKEKNLYLSCVLKDDKPTLQLESVDPKNYPKKKMEKRFVFNKIEINNKLEF<br>ESAQFPNWYISTSQAENMPVFLGGTKGGQDITDFTMQFVSS
155	1f21	MLKQVEIFTDGSALGNPGPGGYGAILRYRGREKTFSAGYTRTTNNRMELMAAIVA<br>LEALKEHAEVILSTDSQYVRQGITQWIHNWKKRGWKTADKKPVKNVDLWQRLDA<br>ALGQHQIKWEWVKGHAGHPENERADELARAAAMNPTLEDTGYQVEV
158	1am7	MVEINNQRKAFLDMLAWSEGTDNGRQKTRNHGYDVIVGGELFTDYSDHPRKLVT<br>LNPKLKSTGAGRYQLLSRWWDAYRKQLGLKDFSPKSQDAVALQQIKERGALPMID<br>RGDIRQAIDRCSNIWASLPGAGYGQFEHKADSLIAKFKEAGGTVREIDV
159	5dfr	MISLIAALAVDRVIGMENAMPWNLPADLAWFKRNTLDKPVIMGRHTWESIGRPLP<br>GRKNIILSSQPGTDDRVTWVKSVDEAIAACGDVPEIMVIGGGRVYEQFLPKAQKLY<br>LTHIDAEVEGDTHFPDYEPDDWESVFSEFHDADAQNSHSYCFEILERR
164	2lzm	MNIFEMLRIDEGLRLKIYKDTEGYYTIGIGHLLTKSPSLNAAKSELDKAIGRNTNGVI<br>TKDEAEKLFNQDVDAAVRGILRNAKLKPVYDSLDAVRRAALINMVFQMGETGVA<br>GFTNSLRMLQQKRWDEAAVNLAKSRWYNQTPNRAKRVITTFRTGTWDAYKNL

Supplementary section S2: External Validation Dataset

The predictive performance and generalization ability of LMEFold were validated on two external datasets: ubiquitin (PDB: 1UBQ) and the PF-HDX-EvalSet.

Table S2 Protein Sequence Related to Model Generalization Ability Validation Based on ubiquitin (PDB ID: 1UBQ)

1UBQ	MQIFVKTLTGKTITLEVEPSDTIENVKAKIQDKEGIPPDQQRLIFAGKQLEDGRTLSDYN IQKESTLHLVLRLRGG<br>0011110000001001100010100101110000000000110110000100001100000000101001000000

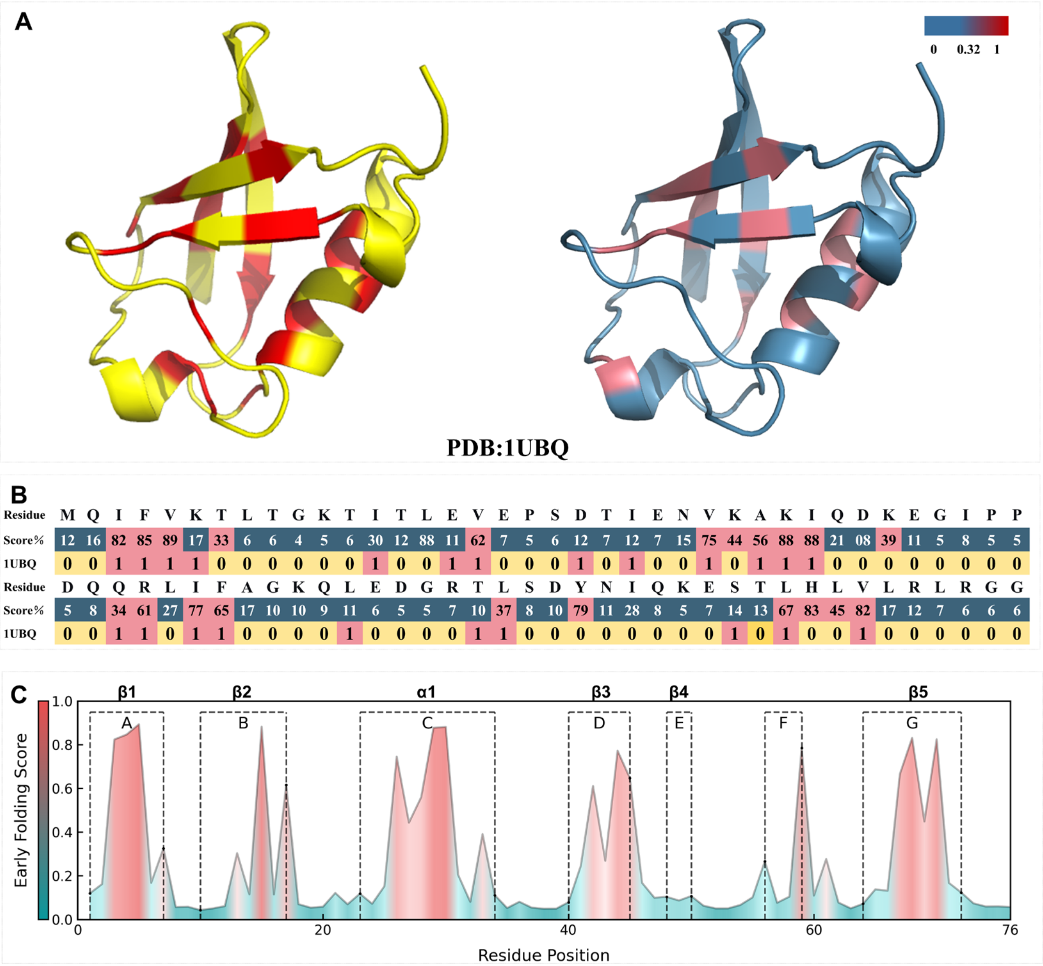

Figure S1 Structural and sequence-level consistency analysis of early-folding signals in ubiquitin (PDB: 1UBQ).

(A) Spatial distribution of experimentally identified early-folding residues mapped onto the three-dimensional structure of ubiquitin (left, in red), alongside the structural mapping of LMEFold-predicted early-folding probabilities (right; color gradient from blue to red indicates increasing probability).

(B) Residue-level correspondence between LMEFold predictions and experimental annotations. The top panel displays the amino acid sequence, the middle panel shows the residue-wise early-folding probabilities predicted by LMEFold, and the bottom panel indicates the experimental labels.

(C) Distribution of LMEFold-predicted early-folding probabilities along the protein sequence. The annotated segments (A–G) correspond to the seven major secondary-structure regions.

Table S3 Hydrogen-Deuterium Exchange-Nuclear Magnetic Resonance Experimentally Validated Protection Factor (PF) Annotated Dataset

1SNO											
Residue	Reported log(PF)	Log(PF)	Amino Acid	Residue	Reported log(PF)	Log(PF)	Amino Acid	Residue	Reported log(PF)	Log(PF)	Amino Acid
1	0.43	0.43	ALA	57	5.73	5.73	GLU	96	7.6	7.6	GLY
2	0.38	0.38	THR	58	6.7	6.7	ALA	97	7.13	7.13	LYS
3	0.19	0.19	SER	59	6.73	6.73	SER	98	7.36	7.36	MET
4	0.88	0.88	THR	60	6.57	6.57	ALA	99	7.23	7.23	VAL
5	3.9	3.9	LYS	61	6.97	6.97	PHE	100	7.17	7.17	ASN
7	4.95	4.95	LEU	62	7.2	7.2	THR	101	7.38	7.38	GLU
8	4.37	4.37	HIS	63	2.35	2.35	LYS	102	7.12	7.12	ALA
9	1.37	1.37	LYS	64	3.95	3.95	LYS	103	6.57	6.57	LEU
10	5.04	5.04	GLU	65	1.7	1.7	MET	104	7.04	7.04	VAL
11	4.19	4.19	PRO	66	3.79	3.79	VAL	105	5.77	5.77	ARG
12	1.01	1.01	ALA	67	1.58	1.58	GLU	106	5.9	5.9	GLN
14	4.16	4.16	LEU	68	7.55	7.55	ASN	107	4.38	4.38	GLY
15	1.33	1.33	ILE	69	5.78	5.78	ALA	114	1.7	1.7	VAL
16	4.6	4.6	LYS	70	7.65	7.65	LYS	115	2.89	2.89	TYR
17	4.68	4.68	ALA	71	3.88	3.88	LYS	117	4.68	4.68	PRO
18	4.99	4.99	ILE	72	4.61	4.61	ILE	118	0.2	0.2	ASN
19	6.71	6.71	ASP	75	0.06	0.06	GLU	119	1.25	1.25	ASN
20	7.55	7.55	GLY	77	2.93	2.93	ASP	120	6.08	6.08	THR
21	6.86	6.86	ASP	78	4.35	4.35	LYS	121	6.29	6.29	HIS
22	4.6	4.6	THR	81	2.75	2.75	ARG	122	4.24	4.24	GLU
23	0.49	0.49	VAL	82	4.61	4.61	THR	123	4.76	4.76	GLN
24	1.17	1.17	LYS	83	4.91	4.91	ASP	125	6.05	6.05	LEU
25	5.17	5.17	LEU	84	5.08	5.08	LYS	126	4.79	4.79	ARG
27	6.96	6.96	TYR	85	7.49	7.49	TYR	127	6.52	6.52	LYS
28	1.09	1.09	LYS	86	7.66	7.66	GLY	128	6.21	6.21	SER
29	7.48	7.48	GLY	87	7.51	7.51	ARG	129	5.52	5.52	GLU
30	7.62	7.62	GLN	88	7.53	7.53	GLY	130	5.02	5.02	ALA
31	6.37	6.37	PRO	89	7.64	7.64	LEU	131	5.06	5.06	GLN
32	7.3	7.3	MET	90	4.42	4.42	ALA	132	5.23	5.23	ALA
34	5.95	5.95	PHE	91	1.26	1.26	TYR	133	2.57	2.57	LYS
35	4.77	4.77	ARG	92	5.74	5.74	ILE	134	5.05	5.05	LYS
53	1.92	1.92	LYS	93	0.28	0.28	TYR	135	4.8	4.8	GLU
54	3.08	3.08	TYR	94	7.32	7.32	ALA	136	4.5	4.5	LYS
56	4.44	4.44	PRO	95	7.48	7.48	ASP				

3BLG											
Resi	Reported	Log(PF)	Amino	Resi	Reported	Log(P	Amino	Resi	Reported	Log(P	Amino

due	raw PF		Acid	due	raw PF	F)	Acid	due	raw PF	F)	Acid
13	50	1.699	GLN	57	3500	3.544	LEU	103	17000	4.230	LEU
14	140	2.146	LYS	58	2870	3.458	LEU	104	38000	4.580	LEU
15	15700	4.196	VAL	59	20100	4.303	GLN	105	50000	4.699	PHE
16	3200	3.505	ALA	61	50	1.699	TRP	106	25000	4.398	CYS
17	200	2.301	GLY	62	350	2.544	GLU	107	50000	4.699	MET
19	6600	3.820	TRP	65	710	2.851	GLU	108	50000	4.699	GLU
20	36200	4.559	TYR	67	5100	3.708	ALA	109	2810	3.449	ASN
21	50	1.699	SER	71	1460	3.164	ILE	112	440	2.643	GLU
23	5500	3.740	ALA	73	2230	3.348	ALA	115	48600	4.687	GLN
24	17500	4.243	MET	74	1520	3.182	GLU	117	360	2.556	LEU
25	6900	3.839	ALA	78	870	2.940	ILE	118	50000	4.699	VAL
27	800	2.903	SER	81	3560	3.551	VAL	119	44500	4.648	CYS
31	100	2.000	LEU	82	3160	3.500	PHE	120	50000	4.699	GLN
32	1570	3.196	LEU	83	620	2.792	LYS	121	9930	3.997	CYS
34	180	2.255	ALA	84	7490	3.874	ILE	122	1470	3.167	LEU
37	80	1.903	ALA	89	660	2.820	GLU	123	26500	4.423	VAL
41	1190	3.076	VAL	90	2170	3.336	ASN	127	850	2.929	GLU
42	120	2.079	TYR	91	50000	4.699	LYS	136	50000	4.699	PHE
43	9530	3.979	VAL	92	12700	4.104	VAL	137	640	2.806	ASP
45	50	1.699	GLU	93	10700	4.029	LEU	139	3140	3.497	ALA
46	1190	3.076	LEU	94	13700	4.137	VAL	140	3930	3.594	LEU
47	210	2.322	LYS	95	50000	4.699	LEU	141	650	2.813	LYS
49	80	1.903	THR	96	280	2.447	ASP	143	1030	3.013	LEU
52	320	2.505	GLY	98	710	2.851	ASP	145	170	2.230	MET
54	22400	4.350	LEU	99	180	2.255	TYR	147	620	2.792	ILE
55	610	2.785	GLU	100	250	2.398	LYS	149	750	2.875	LEU
56	50000	4.699	ILE	102	3000	3.477	TYR				

1A2P											
Residue	Reported raw PF	Log(PF)	Amino Acid	Residue	Reported raw PF	Log(PF)	Amino Acid	Residue	Reported raw PF	Log(PF)	Amino Acid
8	28000	4.447	ASP	34	5700	3.756	GLY	73	420000	5.623	GLU
9	480000	5.681	GLY	42	21000	4.322	LEU	74	210000	5.322	ALA
10	120000	5.079	VAL	43	30000	4.477	ALA	85	410000	5.613	SER
11	370000	5.568	ALA	44	1000000	6.000	ASP	86	530000	5.724	ASP
12	570000	5.756	ASP	47	690000	5.839	PRO	87	530000	5.724	ARG
13	680000	5.833	TYR	48	1100000	6.041	GLY	88	490000	5.690	ILE
14	150000	5.176	LEU	49	240000	5.380	LYS	89	1400000	6.146	LEU
15	250000	5.398	GLN	50	600000	5.778	SER	92	28000	4.447	SER
23	570000	5.756	ASN	51	1200000	6.079	ILE	93	360000	5.556	ASP
24	200000	5.301	TYR	54	310000	5.491	ASP	95	940000	5.973	LEU

28	690000	5.839	SER	69	60000	4.778	ARG	96	860000	5.934	ILE
29	300000	5.477	GLU	70	720000	5.857	THR	97	490000	5.690	TYR
31	310000	5.491	GLN	71	360000	5.556	TRP	105	83000	4.919	THR
33	120000	5.079	LEU	72	1400000	6.146	ARG				

1FS3				1AYL			
Residue	Reported raw PF	Log(PF)	Amino Acid	Residue	Reported ln(PF)	Log(PF)	Amino Acid
10	174000	5.241	ARG	9	8	3.474	PRO
11	17300000	7.238	GLN	10	9.2	3.995	GLN
14	185000	5.267	ASP	11	10.2	4.429	GLU
29	794000	5.900	MET	16	11.5	4.993	GLY
30	24200	4.384	MET	17	11.6	5.037	ILE
31	35000	4.544	LYS	18	11.2	4.863	SER
34	23500	4.371	ASN	21	8.5	3.691	HIS
43	4570	3.660	VAL	23	6	2.605	ILE
44	493000	5.693	ASN	37	6.1	2.649	LEU
48	7410000	6.870	HIS	38	7.5	3.257	ASP
49	444000	5.647	GLU	41	6	2.605	LEU
56	2260000	6.354	ALA	42	10.3	4.472	THR
59	1020000	6.009	SER	53	3.5	1.520	GLY
60	993000	5.997	GLN	54	3.6	1.563	ALA
61	300000	5.477	LYS	55	4.4	1.911	VAL
65	35100	4.545	CYS	67	8.1	3.517	PRO
72	28600000	7.456	CYS	69	8.8	3.821	ASP
83	127000	5.104	ASP	72	9	3.908	ILE
84	14500000	7.161	CYS	73	9.5	4.125	VAL
91	8620	3.936	LYS	74	9	3.908	ARG
97	16800	4.225	TYR	75	8.8	3.821	ASP
98	4440000	6.647	LYS	76	9.9	4.299	ASP
100	2050000	6.312	THR	77	9.8	4.255	THR
102	4310000	6.634	ALA	78	8	3.474	THR
111	1710000	6.233	GLU	85	6.8	2.953	ALA

1BTA				1TM1			
Residue	Reported raw PF	Log(PF)	Amino Acid	Residue	Reported ln(PF)	Log(PF)	Amino Acid
8	15300	4.185	GLU	8	8.1	3.517	LEU
11	17800	4.250	ARG	9	9.9	4.299	VAL
16	2800	3.447	LEU	13	7.2	3.126	VAL
23	4700	3.672	GLU	16	7.1	3.083	ALA
34	3100	3.491	LEU	17	6.6	2.866	LYS
38	5220	3.718	TRP	18	8.2	3.561	LYS

39	3200	3.505	ASP	22	9.5	4.125	GLN
44	5550	3.744	TRP	27	6.7	2.909	ALA
45	4400	3.643	VAL	28	8.2	3.561	GLN
49	65900	4.819	LEU	52	8.5	3.691	ASP
50	4200	3.623	VAL	56	8.4	3.647	ASN
52	3300	3.519	GLU	58	9	3.908	ALA
72	8200	3.914	GLN	59	10.5	4.559	GLN
78	21400	4.330	LYS	63	7.4	3.213	VAL
87	7200	3.857	ILE				

1UBQ											
Residue	Reported raw PF	Log(PF)	Amino Acid	Residue	Reported raw PF	Log(PF)	Amino Acid	Residue	Reported raw PF	Log(PF)	Amino Acid
2	22	1.342	GLN	26	440000	5.643	VAL	50	670	2.826	LEU
3	44000	4.643	ILE	28	11000	4.041	ALA	55	3400	3.531	THR
4	42000	4.623	PHE	29	21000	4.322	LYS	56	27000	4.431	LEU
5	21000	4.322	VAL	30	180000	5.255	ILE	57	330	2.519	SER
6	4100	3.613	LYS	31	2800	3.447	GLN	58	150	2.176	ASP
7	30000	4.477	THR	32	30	1.477	ASP	59	13000	4.114	TYR
12	150	2.176	THR	36	120	2.079	ILE	60	60	1.778	ASN
13	6500	3.813	ILE	40	92	1.964	GLN	61	2400	3.380	ILE
15	71	1.851	LEU	41	1000	3.000	GLN	65	1200	3.079	SER
16	330	2.519	GLU	42	2000	3.301	ARG	67	760	2.881	LEU
17	12000	4.079	VAL	44	42000	4.623	ILE	68	1700	3.230	HIS
22	39	1.591	THR	45	1500	3.176	PHE	69	8200	3.914	LEU
23	17000	4.230	ILE	48	2300	3.362	LYS	70	2900	3.462	VAL
25	1400	3.146	ASN	49	66	1.820	GLN				

1CYE											
Residue	Reported log(PF)	Log(PF)	Amino Acid	Residue	Reported log(PF)	Log(PF)	Amino Acid	Residue	Reported log(PF)	Log(PF)	Amino Acid
8	4.3	4.3	PHE	36	4.1	4.1	ALA	86	3.8	3.8	VAL
9	4	4	LEU	38	3.6	3.6	ASP	87	4	4	THR
10	3.8	3.8	VAL	42	4.2	4.2	ALA	107	3.3	3.3	VAL
11	3.8	3.8	VAL	43	3.8	3.8	LEU	109	3.8	3.8	LYS
12	4	4	ASP	53	4.4	4.4	PHE	116	3.7	3.7	LEU
22	4.3	4.3	ARG	54	4.1	4.1	VAL	117	3.7	3.7	GLU
24	4.8	4.8	LEU	55	3.8	3.8	ILE	118	3.4	3.4	GLU
25	4.8	4.8	LEU	56	4.5	4.5	SER	119	4	4	LYS
26	4.7	4.7	LYS	71	4.2	4.2	THR	120	4	4	LEU
27	4	4	GLU	72	3.8	3.8	ILE	121	4.4	4.4	ASN
28	4.3	4.3	LEU	83	3.9	3.9	VAL	122	3.8	3.8	LYS

30	4.4	4.4	PHE	84	3.9	3.9	LEU	123	3.7	3.7	ILE
34	3.7	3.7	GLU	85	4	4	MET				

2ETL											
Resi due	Reported log(PF)	Log(PF)	Amino Acid	Resi due	Reported log(PF)	Log(PF)	Amino Acid	Resi due	Reported log(PF)	Log(PF)	Amino Acid
14	5.07	5.07	ASN	95	5.52	5.52	CYS	168	6.39	6.39	ILE
16	5.94	5.94	GLU	96	5.13	5.13	GLY	171	6.67	6.67	ASN
17	6.03	6.03	MET	97	6.49	6.49	THR	172	6.6	6.6	ASN
20	4.91	4.91	LYS	98	7.16	7.16	ILE	174	6.43	6.43	ASP
22	4.51	4.51	LEU	100	6.83	6.83	LEU	175	5.82	5.82	GLY
26	4.57	4.57	GLY	101	6.98	6.98	ILE	176	5.47	5.47	HIS
29	5.64	5.64	GLY	103	7.11	7.11	ALA	181	6.12	6.12	ASP
35	5.16	5.16	ASP	115	4.42	4.42	ASP	183	5.73	5.73	ARG
48	7.16	7.16	PRO	118	6.36	6.36	VAL	185	6.45	6.45	PRO
49	6.13	6.13	ALA	129	6.71	6.71	MET	196	5.66	5.66	ASP
50	5.58	5.58	PRO	130	4.98	4.98	SER	197	6.33	6.33	THR
52	5.9	5.9	CYS	131	4.74	4.74	PRO	200	5.46	5.46	LYS
53	6.31	6.31	ALA	133	6.27	6.27	ASP	202	7.34	7.34	ALA
60	5.63	5.63	LEU	134	5.82	5.82	ARG	217	5.81	5.81	VAL
80	5.21	5.21	VAL	164	5.4	5.4	ASN	218	6.45	6.45	ARG
84	6.23	6.23	VAL	165	5.96	5.96	PHE	219	6.28	6.28	PHE
94	4.93	4.93	SER	166	7.02	7.02	HIS	220	6.48	6.48	SER

Table S4 Comparison of Pearson Correlation Coefficients Between Early Folding Prediction Scores (LMEFold vs. EFoldMine) and log(PF)

ID	LMEFold	EFoldMine
1A2P	0.208	-0.079
1AYI	0.049	0.007
1BTA	0.169	-0.139
1CYE	0.186	0.230
1FS3	0.264	0.259
1SNO	0.589	0.496
1TM1	-0.010	0.110
1UBO	0.656	0.496
2ETL	0.364	0.214
3BLG	0.585	0.473
Mean	**0.306**	**0.207**
Pooled r	**0.168**	**0.106**

Supplementary section S3: Parameter Optimization

In each iteration of the outer loop, the average AUC across the inner validation folds was computed for every hyperparameter configuration. The configuration yielding the highest mean AUC was selected as the final setting for the corresponding LOOCV iteration. In cases where multiple configurations achieved identical mean AUC values, the configuration with the larger batch size was preferred to ensure greater gradient stability and training efficiency. This selection strategy guarantees that hyperparameter optimization is strictly confined within each fold, while preserving the independence of the final generalization performance assessment.

The Leave-One-Out method was used to train the model on 30 datasets, resulting in 27 validation sets. The 27 validation sets are numbered sequentially, and for each validation set, parameter optimization is carried out using the 5-fold cross-validation method. Table S5 presents the results of parameter optimization on the validation set 9PCY_val.

Table S5 LMEFold parameter optimization results on 9PCY_val

bz	dp	lr	AUC	Accuracy	F1-Score	Recall	Precision	Specificity
1	0.01	1e-5	0.712	0.777	0.337	0.449	0.371	0.843
	0.05	1e-5	0.712	0.777	0.337	0.449	0.371	0.843
	0.1	1e-5	0.712	0.777	0.337	0.449	0.371	0.843
	0.2	1e-5	0.712	0.777	0.337	0.449	0.371	0.843
	0.01	2e-5	0.78	0.802	0.393	0.496	0.379	0.862
	0.05	2e-5	0.78	0.802	0.393	0.496	0.379	0.862
	0.1	2e-5	0.78	0.802	0.393	0.496	0.379	0.862
	0.2	2e-5	0.78	0.802	0.393	0.496	0.379	0.862
	0.1	5e-5	0.837	0.782	0.459	0.678	0.372	0.806
	**0.2**	**5e-5**	**0.837**	**0.782**	**0.459**	**0.678**	**0.372**	**0.806**
2	0.01	1e-5	0.702	0.761	0.327	0.45	0.304	0.821
	0.05	1e-5	0.702	0.761	0.327	0.45	0.304	0.821
	0.1	1e-5	0.702	0.761	0.327	0.45	0.304	0.821
	0.2	1e-5	0.702	0.761	0.327	0.45	0.304	0.821
	0.1	2e-5	0.777	0.798	0.371	0.476	0.386	0.863
	0.2	2e-5	0.777	0.798	0.371	0.476	0.386	0.863
	0.1	5e-5	0.823	0.787	0.425	0.592	0.374	0.828
	0.2	5e-5	0.823	0.787	0.425	0.592	0.374	0.828
4	0.01	1e-5	0.674	0.739	0.296	0.411	0.296	0.802
	0.05	1e-5	0.674	0.739	0.296	0.411	0.296	0.802
	0.1	1e-5	0.674	0.739	0.296	0.411	0.296	0.802
	0.2	1e-5	0.674	0.739	0.296	0.411	0.296	0.802
	0.1	2e-5	0.738	0.738	0.28	0.441	0.278	0.803
	0.2	2e-5	0.738	0.738	0.28	0.441	0.278	0.803
	0.1	5e-5	0.783	0.787	0.381	0.51	0.361	0.842
	0.2	5e-5	0.783	0.787	0.381	0.51	0.361	0.842

Table S6 Sensitivity Analysis of Class Weight (w) on Validation Set under Severe Class Imbalance for Early Folding Residue Prediction

Weight	AUC	PR-AUC	Accuracy	F1-Score	Recall	Precision	Specificity
1.000	0.820 ± 0.164	0.506 ± 0.113	0.861 ± 0.172	0.309 ± 0.184	0.241 ± 0.175	0.577 ± 0.240	0.972 ± 0.194
3.000	0.822 ± 0.055	0.545 ± 0.078	0.829 ± 0.036	0.482 ± 0.060	0.535 ± 0.138	0.467 ± 0.089	0.883 ± 0.058
5.000	0.843 ± 0.045	0.558 ± 0.078	0.765 ± 0.079	0.479 ± 0.068	0.701 ± 0.158	0.392 ± 0.106	0.779 ± 0.113
7.000	0.837 ± 0.049	0.543 ± 0.089	0.808 ± 0.039	0.492 ± 0.057	0.626 ± 0.156	0.432 ± 0.084	0.844 ± 0.068

Supplementary section S4: Comparison of Methods

To comprehensively evaluate the performance of LMEFold when using different pretrained large language models as feature extractors, we systematically benchmarked four representative models and a dedicated predictor: ESM2-t30, ESM2-t12, ProtBERT, ProtT5, and EFoldMine. Tables S7-S11 present the leave-one-out cross-validation results of these models on the early folding dataset. During data preprocessing, sequence clustering was performed using a strict 25% sequence identity threshold to define homology-aware outer folds. 30 proteins were grouped into 27 mutually exclusive clusters (e.g., [1hel, 1lz1, 2eql] formed fold 8 and [1mbc, 1ymb] formed fold 13). Proteins within the same cluster were held out together during validation, and all 30 protein-level results were fully retained and included when calculating the summary performance metrics.

Table S7 ESM2-t30 leave-one-out cross-validation method results (parameter combination: dp, lr, bz)

Fold	ID	Parameter	AUC	Accuracy	F1 Score	Recall	Precision	Specificity
1	1a64	(0.2,5e-5,1)	0.803	0.784	0.4	0.368	0.438	0.885
2	1am7	(0.2,2e-5,1)	0.753	0.81	0.318	0.241	0.467	0.938
3	1bdd	(0.2,5e-5,1)	0.873	0.817	0.703	0.65	0.765	0.9
4	1coe	(0.2,5e-5,1)	0.696	0.774	0.125	0.167	0.1	0.839
5	1e3y	(0.2,5e-5,1)	0.869	0.798	0.462	0.375	0.6	0.925
6	1f21	(0.2,5e-5,1)	0.817	0.884	0.308	0.571	0.211	0.899
7	1hce	(0.2,5e-5,1)	0.826	0.737	0.34	0.8	0.216	0.731
	1hel	(0.2,5e-5,1)	0.888	0.775	0.453	0.333	0.706	0.946
8	1lzl	(0.2,5e-5,1)	0.815	0.877	0.429	0.462	0.4	0.923
	2eql	(0.2,5e-5,1)	0.891	0.876	0.429	0.6	0.333	0.899
9	1hrc	(0.2,5e-5,1)	0.875	0.875	0.519	0.438	0.636	0.955
10	1hrh	(0.2,5e-5,1)	0.664	0.846	0.167	0.111	0.333	0.964
11	1i1b	(0.2,5e-5,1)	0.821	0.739	0.474	0.857	0.327	0.72
12	1joo	(0.2,5e-5,1)	0.788	0.819	0.229	0.444	0.154	0.843
13	1mbc	(0.2,5e-5,1)	0.941	0.791	0.429	1	0.273	0.773
	1ymb	(0.2,5e-5,1)	0.897	0.784	0.441	0.867	0.295	0.775
14	1omu	(0.2,5e-5,1)	0.861	0.821	0.167	0.25	0.125	0.865
15	1onc	(0.2,5e-5,1)	0.815	0.731	0.333	0.226	0.636	0.945
16	1pga	(0.2,5e-5,1)	0.797	0.679	0.5	0.346	0.9	0.967
17	1rbx	(0.2,5e-5,1)	0.795	0.839	0.412	0.5	0.35	0.882
18	1rg8	(0.2,5e-5,1)	0.803	0.736	0.413	0.342	0.52	0.882
19	1ygw	(0.2,5e-5,2)	0.827	0.827	0.438	0.538	0.368	0.868
20	2abd	(0.2,5e-5,2)	0.761	0.791	0.357	0.455	0.294	0.84
21	2crt	(0.2,2e-5,1)	0.729	0.783	0.381	0.333	0.444	0.896
22	2lzm	(0.2,5e-5,1)	0.83	0.854	0.294	0.714	0.185	0.86
23	2ptl	(0.2,5e-5,1)	0.875	0.769	0.55	0.917	0.393	0.742
24	2vil	(0.2,5e-5,1)	0.893	0.802	0.419	0.29	0.75	0.968
25	5dfr	(0.2,5e-5,1)	0.91	0.849	0.25	0.8	0.148	0.851
26	5pti	(0.2,5e-5,1)	0.866	0.845	0.471	0.571	0.4	0.882
27	9pcy	(0.2,5e-5,1)	0.957	0.889	0.476	0.833	0.333	0.892
Mean	\	\	0.831	0.807	0.390	0.513	0.403	0.875
St.dev	\	\	0.068	0.051	0.123	0.244	0.207	0.070

Table S8 ESM2-t12 leave-one-out cross-validation method results

Fold	ID	Parameter	AUC	Accuracy	F1	Recall	Precision	Specificity
1	1a64	(0.2,5e-5,1)	0.776	0.814	0.400	0.316	0.545	0.936
2	1am7	(0.2,5e-5,2)	0.672	0.778	0.314	0.276	0.364	0.891
3	1bdd	(0.2,5e-5,1)	0.790	0.717	0.667	0.850	0.548	0.650
4	1coe	(0.2,5e-5,1)	0.682	0.532	0.216	0.667	0.129	0.518
5	1e3y	(0.2,5e-5,1)	0.823	0.750	0.519	0.583	0.467	0.800
6	1f21	(0.2,5e-5,1)	0.834	0.800	0.279	0.857	0.167	0.797
7	1hce	(0.2,5e-5,1)	0.842	0.763	0.364	0.800	0.235	0.759
	1hel	(0.2,5e-5,1)	0.899	0.822	0.610	0.500	0.783	0.946
8	1lzl	(0.2,5e-5,1)	0.828	0.862	0.357	0.385	0.333	0.915
	2eql	(0.2,5e-5,1)	0.787	0.853	0.296	0.400	0.235	0.891
9	1hrc	(0.2,5e-5,1)	0.921	0.817	0.558	0.750	0.444	0.830
10	1hrh	(0.2,5e-5,1)	0.731	0.838	0.160	0.111	0.286	0.955
11	1i1b	(0.2,5e-5,1)	0.852	0.732	0.481	0.905	0.328	0.705
12	1joo	(0.2,5e-5,1)	0.744	0.705	0.185	0.556	0.111	0.714
13	1mbc	(0.2,5e-5,1)	0.908	0.739	0.355	0.917	0.220	0.723
	1ymb	(0.2,5e-5,1)	0.870	0.876	0.424	0.467	0.389	0.920
14	1omu	(0.2,5e-5,1)	0.861	0.786	0.333	0.750	0.214	0.788
15	1onc	(0.2,5e-5,1)	0.793	0.740	0.509	0.452	0.583	0.863
16	1pga	(0.2,5e-5,1)	0.895	0.696	0.564	0.423	0.846	0.933
17	1rbx	(0.2,5e-5,1)	0.805	0.726	0.320	0.571	0.222	0.745
18	1rg8	(0.2,5e-5,1)	0.812	0.779	0.551	0.500	0.613	0.882
19	1ygw	(0.2,5e-5,4)	0.697	0.663	0.314	0.615	0.211	0.670
20	2abd	(0.2,5e-5,2)	0.766	0.756	0.222	0.273	0.188	0.827
21	2crt	(0.2,2e-5,1)	0.684	0.600	0.368	0.583	0.269	0.604
22	2lzm	(0.2,5e-5,1)	0.756	0.793	0.190	0.571	0.114	0.803
23	2ptl	(0.2,5e-5,1)	0.823	0.782	0.485	0.667	0.381	0.803
24	2vil	(0.2,5e-5,1)	0.880	0.833	0.677	0.710	0.647	0.874
25	5dfr	(0.2,5e-5,1)	0.900	0.767	0.178	0.800	0.100	0.766
26	5pti	(0.2,5e-5,1)	0.798	0.793	0.500	0.857	0.353	0.784
27	9pcy	(0.2,5e-5,1)	0.928	0.657	0.261	1.000	0.150	0.634
Mean	\	\	0.812	0.759	0.389	0.604	0.349	0.798
St.dev	\	\	0.073	0.076	0.150	0.220	0.202	0.111

Table S9 ProtBERT leave-one-out cross-validation method results

Fold	ID	Parameter	AUC	Accuracy	F1	Recall	Precision	Specificity
1	1a64	(0.2,1e-5,1)	0.753	0.649	0.414	0.632	0.308	0.654
2	1am7	(0.2,1e-5,1)	0.654	0.816	0.256	0.172	0.500	0.961
3	1bdd	(0.2,1e-5,1)	0.865	0.733	0.429	0.300	0.750	0.950
4	1coe	(0.2,1e-5,1)	0.598	0.629	0.148	0.333	0.095	0.661
5	1e3y	(0.2,1e-5,1)	0.843	0.625	0.506	0.833	0.364	0.562

6	1f21	(0.2,1e-5,1)	0.849	0.916	0.235	0.286	0.200	0.946
7	1hce	(0.2,1e-5,1)	0.823	0.712	0.320	0.800	0.200	0.704
	1hel	(0.2,5e-5,4)	0.869	0.814	0.556	0.417	0.833	0.968
8	1lzl	(0.2,5e-5,4)	0.824	0.869	0.485	0.615	0.400	0.897
	2eql	(0.2,5e-5,4)	0.871	0.853	0.387	0.600	0.286	0.874
9	1hrc	(0.2,1e-5,1)	0.887	0.865	0.300	0.188	0.750	0.989
10	1hrh	(0.2,1e-5,1)	0.637	0.808	0.194	0.167	0.231	0.911
11	1i1b	(0.2,1e-5,1)	0.825	0.660	0.409	0.857	0.269	0.629
12	1joo	(0.2,1e-5,1)	0.758	0.866	0.167	0.222	0.133	0.907
13	1mbc	(0.2,1e-5,1)	0.917	0.804	0.423	0.917	0.275	0.794
	1ymb	(0.2,1e-5,1)	0.863	0.869	0.286	0.267	0.308	0.935
14	1omu	(0.2,1e-5,1)	0.846	0.750	0.364	1.000	0.222	0.731
15	1onc	(0.2,1e-5,1)	0.607	0.510	0.427	0.613	0.328	0.466
16	1pga	(0.2,1e-5,1)	0.815	0.732	0.667	0.577	0.789	0.867
17	1rbx	(0.2,1e-5,1)	0.704	0.685	0.291	0.571	0.195	0.700
18	1rg8	(0.2,1e-5,1)	0.754	0.750	0.462	0.395	0.556	0.882
19	1ygw	(0.2,1e-5,1)	0.730	0.760	0.242	0.308	0.200	0.824
20	2abd	(0.2,1e-5,1)	0.684	0.512	0.323	0.909	0.196	0.453
21	2crt	(0.2,1e-5,1)	0.641	0.750	0.400	0.417	0.385	0.833
22	2lzm	(0.2,1e-5,1)	0.852	0.921	0.235	0.286	0.200	0.949
23	2ptl	(0.2,1e-5,1)	0.900	0.795	0.529	0.750	0.409	0.803
24	2vil	(0.2,1e-5,1)	0.849	0.817	0.693	0.839	0.591	0.811
25	5dfr	(0.2,1e-5,1)	0.948	0.962	0.400	0.400	0.400	0.981
26	5pti	(0.2,1e-5,1)	0.910	0.879	0.632	0.857	0.500	0.882
27	9pcy	(0.2,1e-5,1)	0.934	0.869	0.381	0.667	0.267	0.882
Mean	\	\	0.800	0.773	0.385	0.540	0.371	0.814
St.dev	\	\	0.102	0.113	0.141	0.259	0.203	0.150

Table S10 ProtT5 leave-one-out cross-validation method results

Fold	ID	Parameter	AUC	Accuracy	F1	Recall	Precision	Specificity
1	1a64	(0.2,5e-5,1)	0.702	0.732	0.316	0.316	0.316	0.833
2	1am7	(0.2,5e-5,1)	0.694	0.785	0.393	0.379	0.407	0.876
3	1bdd	(0.2,5e-5,1)	0.859	0.767	0.696	0.800	0.615	0.750
4	1coe	(0.2,5e-5,1)	0.658	0.742	0.273	0.500	0.188	0.768
5	1e3y	(0.2,5e-5,1)	0.840	0.817	0.612	0.625	0.600	0.875
6	1f21	(0.2,2e-5,1)	0.751	0.832	0.278	0.714	0.172	0.838
7	1hce	(0.2,5e-5,1)	0.897	0.746	0.375	0.900	0.237	0.731
	1hel	(0.2,5e-5,1)	0.888	0.798	0.552	0.444	0.727	0.935
8	1lzl	(0.2,5e-5,1)	0.733	0.769	0.211	0.308	0.160	0.821
	2eql	(0.2,5e-5,1)	0.841	0.829	0.353	0.600	0.250	0.849
9	1hrc	(0.2,5e-5,1)	0.743	0.865	0.364	0.250	0.667	0.977
10	1hrh	(0.2,5e-5,1)	0.759	0.862	0.357	0.278	0.500	0.955
11	1i1b	(0.2,5e-5,2)	0.710	0.621	0.356	0.762	0.232	0.598
12	1joo	(0.2,5e-5,1)	0.784	0.926	0.154	0.111	0.250	0.979

13	1mbc	(0.2,5e-5,1)	0.891	0.791	0.360	0.750	0.237	0.794
	1ymb	(0.2,5e-5,1)	0.849	0.830	0.381	0.533	0.296	0.862
14	1omu	(0.2,5e-5,1)	0.894	0.893	0.400	0.500	0.333	0.923
15	1onc	(0.2,5e-5,1)	0.661	0.702	0.392	0.323	0.500	0.863
16	1pga	(0.2,5e-5,1)	0.862	0.661	0.457	0.308	0.889	0.967
17	1rbx	(0.2,5e-5,1)	0.735	0.274	0.211	0.857	0.120	0.200
18	1rg8	(0.2,5e-5,1)	0.766	0.729	0.406	0.342	0.500	0.873
19	1ygw	(0.2,5e-5,1)	0.834	0.846	0.333	0.308	0.364	0.923
20	2abd	(0.2,5e-5,1)	0.777	0.756	0.276	0.364	0.222	0.813
21	2crt	(0.2,5e-5,1)	0.656	0.750	0.118	0.083	0.200	0.917
22	2lzm	(0.2,5e-5,1)	0.926	0.927	0.455	0.714	0.333	0.936
23	2ptl	(0.2,5e-5,1)	0.789	0.769	0.438	0.583	0.350	0.803
24	2vil	(0.2,5e-5,1)	0.883	0.810	0.613	0.613	0.613	0.874
25	5dfr	(0.2,5e-5,1)	0.848	0.874	0.167	0.400	0.105	0.890
26	5pti	(0.2,5e-5,1)	0.924	0.897	0.571	0.571	0.571	0.941
27	9pcy	(0.2,5e-5,1)	0.973	0.899	0.545	1.000	0.375	0.892
Mean	\	\	0.804	0.783	0.380	0.508	0.378	0.842
St.dev	\	\	0.088	0.122	0.142	0.234	0.198	0.147

Table S11 EFoldMine leave-one-out cross-validation method results

Fold	ID	Parameter	AUC	Accuracy	F1	Recall	Precision	Specificity
1	1a64	\	0.635	0.680	0.415	0.579	0.324	0.705
2	1am7	\	0.651	0.741	0.423	0.517	0.357	0.791
3	1bdd	\	0.836	0.767	0.588	0.500	0.714	0.900
4	1coe	\	0.723	0.790	0.381	0.667	0.267	0.804
5	1e3y	\	0.785	0.663	0.493	0.708	0.378	0.650
6	1f21	\	0.955	0.768	0.25	0.857	0.146	0.764
7	1hce	\	0.832	0.881	0.222	0.200	0.250	0.944
	1hel	\	0.848	0.798	0.658	0.694	0.625	0.839
8	1lzl	\	0.881	0.608	0.338	1.000	0.203	0.564
	2eql	\	0.847	0.659	0.290	0.900	0.173	0.639
9	1hrc	\	0.855	0.798	0.533	0.750	0.414	0.807
10	1hrh	\	0.724	0.800	0.480	0.667	0.375	0.821
11	1i1b	\	0.851	0.706	0.430	0.810	0.293	0.689
12	1joo	\	0.787	0.698	0.262	0.889	0.154	0.686
13	1mbc	\	0.904	0.804	0.400	0.833	0.263	0.801
	1ymb	\	0.800	0.621	0.326	0.933	0.197	0.587
14	1omu	\	0.899	0.661	0.240	0.750	0.143	0.654
15	1onc	\	0.728	0.644	0.543	0.710	0.440	0.616
16	1pga	\	0.895	0.857	0.833	0.769	0.909	0.933
17	1rbx	\	0.852	0.831	0.432	0.571	0.348	0.864
18	1rg8	\	0.791	0.750	0.545	0.553	0.538	0.824
19	1ygw	\	0.757	0.721	0.408	0.769	0.278	0.714
20	2abd	\	0.707	0.674	0.300	0.545	0.207	0.693

21	2crt	\	0.628	0.533	0.364	0.667	0.250	0.500
22	2lzm	\	0.857	0.671	0.182	0.857	0.102	0.662
23	2ptl	\	0.795	0.692	0.455	0.833	0.312	0.667
24	2vil	\	0.833	0.849	0.642	0.548	0.773	0.947
25	5dfr	\	0.821	0.660	0.156	1.000	0.085	0.649
26	5pti	\	0.839	0.845	0.571	0.857	0.429	0.843
27	9pcy	\	0.832	0.636	0.250	1.000	0.143	0.613
Mean	\	\	0.805	0.727	0.414	0.731	0.336	0.739
St.dev	\	\	0.081	0.086	0.157	0.179	0.202	0.119

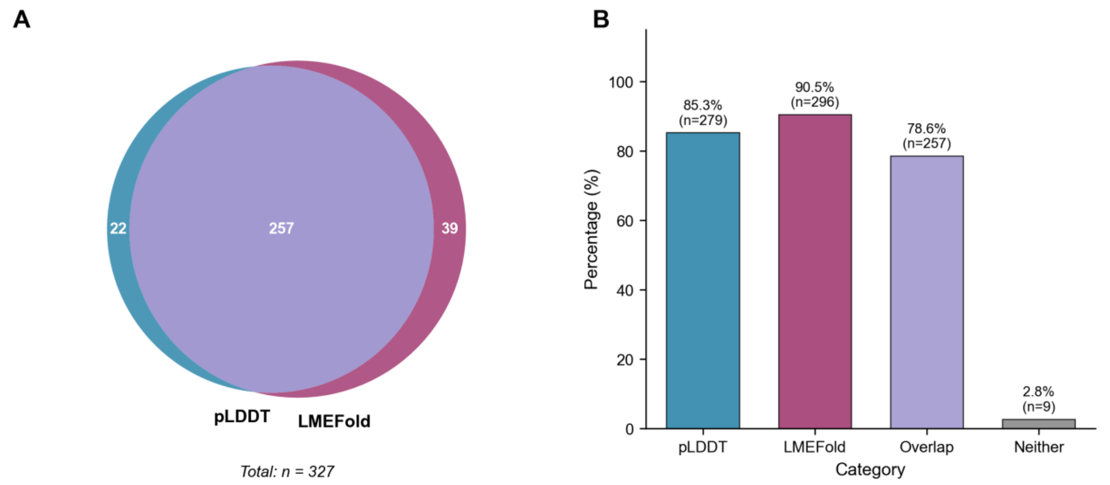

Figure S2 Discriminative advantage of LMEFold over AlphaFold pLDDT in identifying early folding residues. (A) Overlap of early folding residues identified by LMEFold and AlphaFold pLDDT (total n = 327). (B) Percentage of early folding residues identified by LMEFold, AlphaFold pLDDT, their overlap, and residues identified by neither.

Supplementary section S5: Enrichment Analysis of Mutations at EFRs

This section summarizes the mutation enrichment analysis across the ClinVar pathogenicity tiers and MSK-MET pan-cancer cohort (n = 27 types). Figure S3 displays the proportion of EFR mutations across the three pathogenicity groups. Enrichment scores were calculated to compare somatic mutation proportions between early-folding and non-early-folding sites. Statistical significance was assessed via Wilcoxon rank-sum tests. Table S12 details the distribution (Min, Max, Mean, and Quartiles) of enrichment scores for each malignancy, with associated *P*-values indicating the evidence for preferential mutation accumulation at early-folding regions.

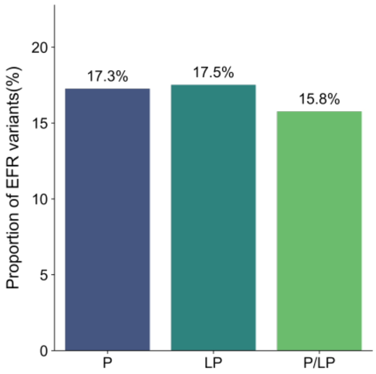

Figure S3 Comparison of EFR variant proportions across ClinVar pathogenicity tiers. The proportions of EFR variants were calculated for three distinct pathogenicity categories: Pathogenic (P), Likely Pathogenic (LP), and Pathogenic/Likely Pathogenic (P/LP).

Table S12 Enrichment scores of 27 cancer types across the MSK-MET cohort

Disease	Min	Max	Mean	Q25	Q50	Q75	*P*-value
Anal Cancer	0.294	6.600	2.360	0.788	1.618	3.752	0.005
Appendiceal Cancer	0.835	23.209	3.361	1.568	2.508	3.069	<0.001
Bladder Cancer	0.169	23.046	2.217	0.811	1.384	2.485	<0.001
Breast Cancer	0.280	23.847	3.801	1.669	2.819	4.994	<0.001
Cervical Cancer	0.525	10.809	2.130	0.979	1.424	2.143	0.007
Colorectal Cancer	0.197	18.890	2.914	1.215	2.011	3.697	<0.001
Endometrial Cancer	0.271	27.917	2.373	0.892	1.549	2.839	<0.001
Esophagogastric Cancer	0.418	31.969	4.137	1.914	3.046	5.255	<0.001
Gastrointestinal Neuroendocrine Tumor	0.795	8.583	3.471	1.590	2.246	4.728	<0.001
Gastrointestinal Stromal Tumor	1.569	11.488	4.790	2.748	5.366	6.298	<0.001
Germ Cell Tumor	1.007	13.954	4.749	2.691	4.449	5.733	<0.001
Head and Neck Cancer	0.251	28.513	3.099	1.084	2.068	3.366	<0.001
Hepatobiliary Cancer	0.240	34.009	4.049	1.979	3.077	5.339	<0.001
Melanoma	0.110	16.758	1.550	0.509	0.800	1.501	0.002
Mesothelioma	0.573	9.919	5.166	2.461	5.877	7.064	0.001
Non-Small Cell Lung Cancer	0.168	32.733	2.551	1.059	1.732	3.025	<0.001
Ovarian Cancer	0.375	26.320	4.718	2.205	4.038	5.911	<0.001
Pancreatic Cancer	0.305	18.812	3.923	2.114	3.040	5.063	<0.001
Prostate Cancer	0.211	27.860	4.830	2.273	3.957	6.391	<0.001
Renal Cell Carcinoma	0.830	15.071	5.369	2.714	4.246	6.787	<0.001
Salivary Gland Cancer	0.870	8.757	4.974	2.519	5.324	7.280	0.004

Skin Cancer, Non-Melanoma	0.181	7.631	1.087	0.409	0.663	0.923	<0.001
Small Bowel Cancer	0.717	16.319	2.589	1.246	1.737	2.574	<0.001
Small Cell Lung Cancer	0.446	24.825	3.064	1.282	1.954	3.131	<0.001
Soft Tissue Sarcoma	0.487	24.289	5.229	2.603	4.236	5.955	<0.001
Thyroid Cancer	0.164	13.826	4.494	2.924	3.858	6.006	<0.001
Uterine Sarcoma	0.735	21.153	5.151	2.042	4.238	5.947	<0.001

Supplementary sectionS6: Log-Rank Test of cohort

ID: Unique identifier for mutation associations. A single UniProt ID (e.g., P04637, corresponding to the tumor suppressor gene *TP53*) indicates mutation analysis at the protein level; the format "UniProt ID_amino acid mutation" (e.g., P04637_R273S) indicates a missense mutation at a single EFR site on a specific protein (e.g., R273S denotes the mutation of arginine to serine at position 273 of the *TP53* protein).
Log-Rank Test *P*-value: Probability value calculated by the Log-Rank test, used to quantify the significance of differences in Kaplan-Meier survival curves between the "mutant group" and "wild-type group" of patients. A smaller *P*-value indicates a stronger association between the mutation and patient prognosis.
Log-Rank Test adjusted *P*-value: Log-Rank test *P*-value after False Discovery Rate (FDR) correction.
Disease: Cancer type corresponding to the mutation-prognosis association, covering major cancer types in the pan-cancer cohort such as endometrial cancer, prostate cancer, and non-small cell lung cancer.
Granularity: Indicator of the refinement of mutation grouping strategies, corresponding to three grouping logics for survival analysis:
① "only_protein": grouping based on whether there is any missense mutation in the entire protein (without distinguishing EFR sites);
② "efr": grouping based on whether there is any missense mutation in the EFR region of the protein (focusing only on EFR sites, without specifying individual sites);
③ "site": grouping based on whether there is a missense mutation at a specific EFR site of the protein (the most refined site-level analysis).

Table S13 Association Between Early Folding Site-Related Missense Mutations and Patient Survival (MSK-MET Cohort)

ID	Mut_n	Wild_n	Log-Rank Test *P*-value	Log-Rank Test adjusted *P*-value	Disease	Granularity
Q86U86	46	4209	0.008	0.151	Non-Small Cell Lung Cancer	only_protein
Q16236	131	4124	0.001	0.034	Non-Small Cell Lung Cancer	only_protein
P51532	195	4060	<0.001	<0.001	Non-Small Cell Lung Cancer	only_protein
P42338	50	3385	0.001	0.008	Colorectal Cancer	only_protein
Q07864	136	3299	0.003	0.015	Colorectal Cancer	only_protein
P26358	90	3345	<0.001	0.001	Colorectal Cancer	only_protein
P22455	62	3373	<0.001	0.005	Colorectal Cancer	only_protein
P35968	101	3334	0.001	0.006	Colorectal Cancer	only_protein
P42345	110	3325	0.001	0.008	Colorectal Cancer	only_protein
A0A075B6H0	231	3204	<0.001	0.002	Colorectal Cancer	only_protein
O15360	76	3359	<0.001	0.002	Colorectal Cancer	only_protein
P16234	80	3355	0.044	0.079	Colorectal Cancer	only_protein

Q9UQQ2	66	3369	0.001	0.008	Colorectal Cancer	only_protein
Q9BXL7	144	3291	<0.001	0.001	Colorectal Cancer	only_protein
Q12879	180	3255	0.035	0.065	Colorectal Cancer	only_protein
Q13315	105	3330	0.019	0.044	Colorectal Cancer	only_protein
P04637	550	1601	<0.001	<0.001	Breast Cancer	only_protein
P08922	24	2127	<0.001	<0.001	Breast Cancer	only_protein
A0A075B6H0	260	812	<0.001	0.004	Melanoma	only_protein
P46100	70	1002	0.030	0.088	Melanoma	only_protein
P04637	453	828	<0.001	<0.001	Endometrial Cancer	only_protein
P48736	62	1219	0.020	0.030	Endometrial Cancer	only_protein
P21359	53	1228	0.001	0.004	Endometrial Cancer	only_protein
Q07864	105	1176	<0.001	0.001	Endometrial Cancer	only_protein
O60674	41	1240	0.019	0.028	Endometrial Cancer	only_protein
P23468	80	1201	<0.001	0.001	Endometrial Cancer	only_protein
P54756	74	1207	0.001	0.004	Endometrial Cancer	only_protein
P60484	347	934	<0.001	<0.001	Endometrial Cancer	only_protein
P08922	69	1212	<0.001	0.001	Endometrial Cancer	only_protein
P06400	35	1246	0.005	0.010	Endometrial Cancer	only_protein
Q14517	69	1212	0.001	0.003	Endometrial Cancer	only_protein
P04637	374	1236	<0.001	<0.001	Prostate Cancer	only_protein
P10275	83	1527	<0.001	<0.001	Prostate Cancer	only_protein
O43791	277	1333	<0.001	<0.001	Prostate Cancer	only_protein
P04637	15	120	0.006	0.006	Mesothelioma	only_protein
Q15831	16	4239	0.012	0.308	Non-Small Cell Lung Cancer	efr
Q86U86	8	4247	0.006	0.308	Non-Small Cell Lung Cancer	efr
Q9NYY3	9	4246	0.015	0.308	Non-Small Cell Lung Cancer	efr
Q16236	8	4247	0.014	0.308	Non-Small Cell Lung Cancer	efr
P03372	6	4249	0.017	0.311	Non-Small Cell Lung Cancer	efr
P17948	26	4229	0.015	0.308	Non-Small Cell Lung Cancer	efr
P51532	20	4235	<0.001	0.022	Non-Small Cell Lung Cancer	efr
O15327	6	1095	0.002	0.073	Bladder Cancer	efr
P42338	11	3424	0.037	0.424	Colorectal Cancer	efr
Q07864	18	3417	0.042	0.424	Colorectal Cancer	efr
P26358	10	3425	0.025	0.424	Colorectal Cancer	efr
P22455	10	3425	0.031	0.424	Colorectal Cancer	efr
O15550	5	3430	0.038	0.424	Colorectal Cancer	efr
O75030	6	3429	0.025	0.424	Colorectal Cancer	efr
P35968	11	3424	0.028	0.424	Colorectal Cancer	efr
P42345	18	3417	0.005	0.424	Colorectal Cancer	efr
P41212	7	3428	0.028	0.424	Colorectal Cancer	efr
A0A075B6H0	40	3395	0.003	0.424	Colorectal Cancer	efr
O15360	9	3426	0.047	0.424	Colorectal Cancer	efr
P08922	24	3411	0.044	0.424	Colorectal Cancer	efr
P16234	18	3417	0.013	0.424	Colorectal Cancer	efr

Q9UQQ2	8	3427	0.041	0.424	Colorectal Cancer	efr
Q9BXL7	15	3420	0.020	0.424	Colorectal Cancer	efr
Q12879	15	3420	0.035	0.424	Colorectal Cancer	efr
Q13315	19	3416	0.021	0.424	Colorectal Cancer	efr
P04637	177	1974	0.001	0.014	Breast Cancer	efr
P08922	7	2144	0.003	0.021	Breast Cancer	efr
A0A075B6H0	43	1029	0.015	0.606	Melanoma	efr
P46100	8	1064	0.039	0.606	Melanoma	efr
P60484	10	1062	0.048	0.606	Melanoma	efr
Q8NEB9	7	1065	0.048	0.606	Melanoma	efr
P04637	143	1138	<0.001	<0.001	Endometrial Cancer	efr
P48736	19	1262	0.039	0.304	Endometrial Cancer	efr
P21359	20	1261	0.006	0.248	Endometrial Cancer	efr
Q07864	38	1243	0.001	0.087	Endometrial Cancer	efr
O60674	13	1268	0.046	0.304	Endometrial Cancer	efr
P23468	15	1266	0.038	0.304	Endometrial Cancer	efr
P54756	14	1267	0.042	0.304	Endometrial Cancer	efr
P60484	67	1214	0.010	0.284	Endometrial Cancer	efr
P08922	20	1261	0.027	0.304	Endometrial Cancer	efr
P06400	14	1267	0.018	0.304	Endometrial Cancer	efr
Q14517	15	1266	0.033	0.304	Endometrial Cancer	efr
P04637	180	829	0.019	0.076	Ovarian Cancer	efr
P04637	141	1469	0.005	0.033	Prostate Cancer	efr
A0A075B6H0	5	1605	0.007	0.033	Prostate Cancer	efr
P10275	34	1576	0.018	0.067	Prostate Cancer	efr
O43791	106	1504	0.001	0.020	Prostate Cancer	efr
Q70Z35	6	755	0.050	0.348	Esophagogastric Cancer	efr
P04637	9	126	<0.001	<0.001	Mesothelioma	efr
P04637_V173L	12	4243	0.032	0.260	Non-Small Cell Lung Cancer	site
P04637_R273S	6	4249	<0.001	0.001	Non-Small Cell Lung Cancer	site
P04637_V157F	50	4205	0.015	0.166	Non-Small Cell Lung Cancer	site
Q15831_W308C	6	4249	0.038	0.260	Non-Small Cell Lung Cancer	site
P04637_A159V	8	4247	0.012	0.166	Non-Small Cell Lung Cancer	site
P04637_R273H	5	1096	0.034	0.205	Bladder Cancer	site
P04637_M237I	14	3421	0.003	0.101	Colorectal Cancer	site
P04626_L755S	7	3428	0.040	0.585	Colorectal Cancer	site
P04637_Y236C	8	2143	0.003	0.021	Breast Cancer	site
P04637_R273H	22	2129	0.041	0.150	Breast Cancer	site
P04637_R273H	32	1249	<0.001	0.006	Endometrial Cancer	site
P04637_R273C	28	1253	0.001	0.006	Endometrial Cancer	site
Q07864_V411L	25	1256	0.008	0.044	Endometrial Cancer	site
P04637_Y163C	6	1003	0.014	0.140	Ovarian Cancer	site
P04637_R273H	8	1602	<0.001	0.001	Prostate Cancer	site

O43791_F102V	19	1591	0.014	0.062	Prostate Cancer	site
P10275_T878A	19	1591	0.003	0.019	Prostate Cancer	site

Note: Only associations with nominal log-rank $P < 0.05$ are listed; statistical significance after multiple-testing correction was determined using the corresponding BH-adjusted *P* values.

Table S14 Selected cases with nominal EFR-level associations but no protein-level association (MSK-MET Cohort)

Disease	ID	EFR *P*-value	EFR adjusted *P*-value	Protein *P*-value	Protein adjusted *P*-value
Non-Small Cell Lung Cancer	Q15831	0.012	0.308	0.258	0.690
Non-Small Cell Lung Cancer	Q9NYY3	0.015	0.308	0.302	0.700
Non-Small Cell Lung Cancer	P03372	0.017	0.311	0.378	0.740
Non-Small Cell Lung Cancer	P17948	0.015	0.308	0.618	0.893
Bladder Cancer	O15327	0.002	0.073	0.862	0.883
Colorectal Cancer	O15550	0.038	0.424	0.513	0.551
Colorectal Cancer	O75030	0.025	0.424	0.672	0.692
Colorectal Cancer	P41212	0.028	0.424	0.155	0.207
Colorectal Cancer	P08922	0.044	0.424	0.272	0.327
Melanoma	P60484	0.048	0.606	0.424	0.589
Melanoma	Q8NEB9	0.048	0.606	0.058	0.112
Ovarian Cancer	P04637	0.019	0.076	0.448	0.897
Prostate Cancer	A0A075B6H0	0.007	0.033	0.420	0.841
Esophagogastric Cancer	Q70Z35	0.050	0.348	0.183	0.428

Note: All *P* values were obtained from the Log-Rank test and adjusted using the BH method.

Supplementary section S7: Kaplan–Meier Survival Analysis

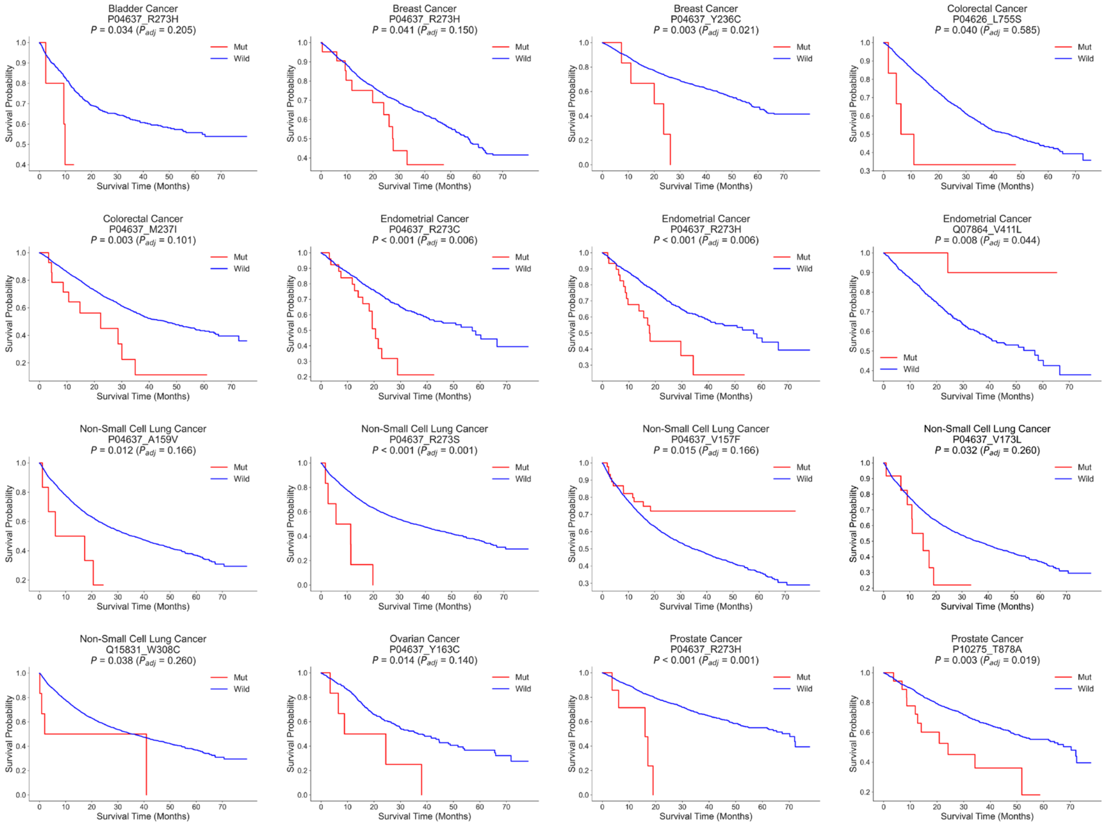

Figure S4. Site-Specific Mutation–Based Survival Analysis

Samples were grouped based on whether mutations occurred at specific target sites. Kaplan-Meier survival curves illustrate the overall survival differences associated with site-specific mutations. P-values were calculated using the Log-Rank test to indicate the potential prognostic impact of these mutations.

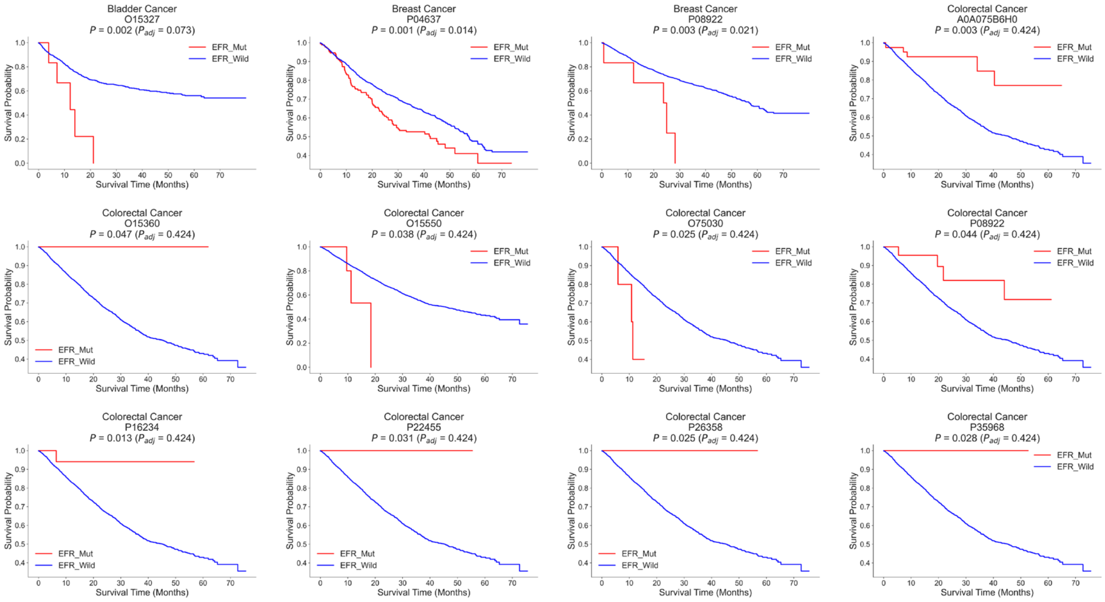

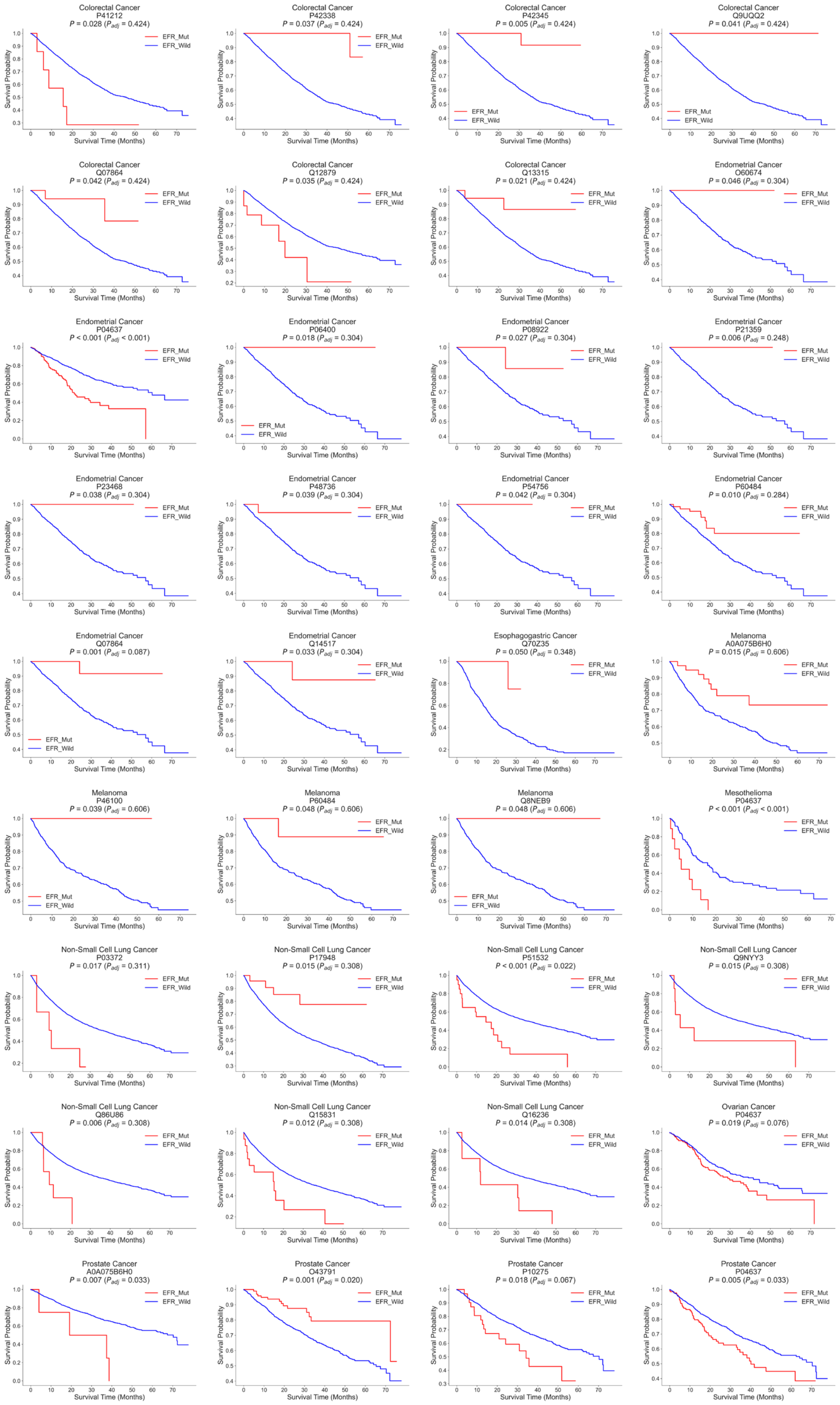

Figure S5. Survival Analysis of Mutations in Early Folding Residues (EFRs)

Samples were grouped based on whether mutations occurred in EFRs. Kaplan-Meier survival curves intuitively demonstrate the association between EFR mutations and patient prognosis. *P*-values were obtained via the

Log-Rank test to quantify the statistical significance of this association.

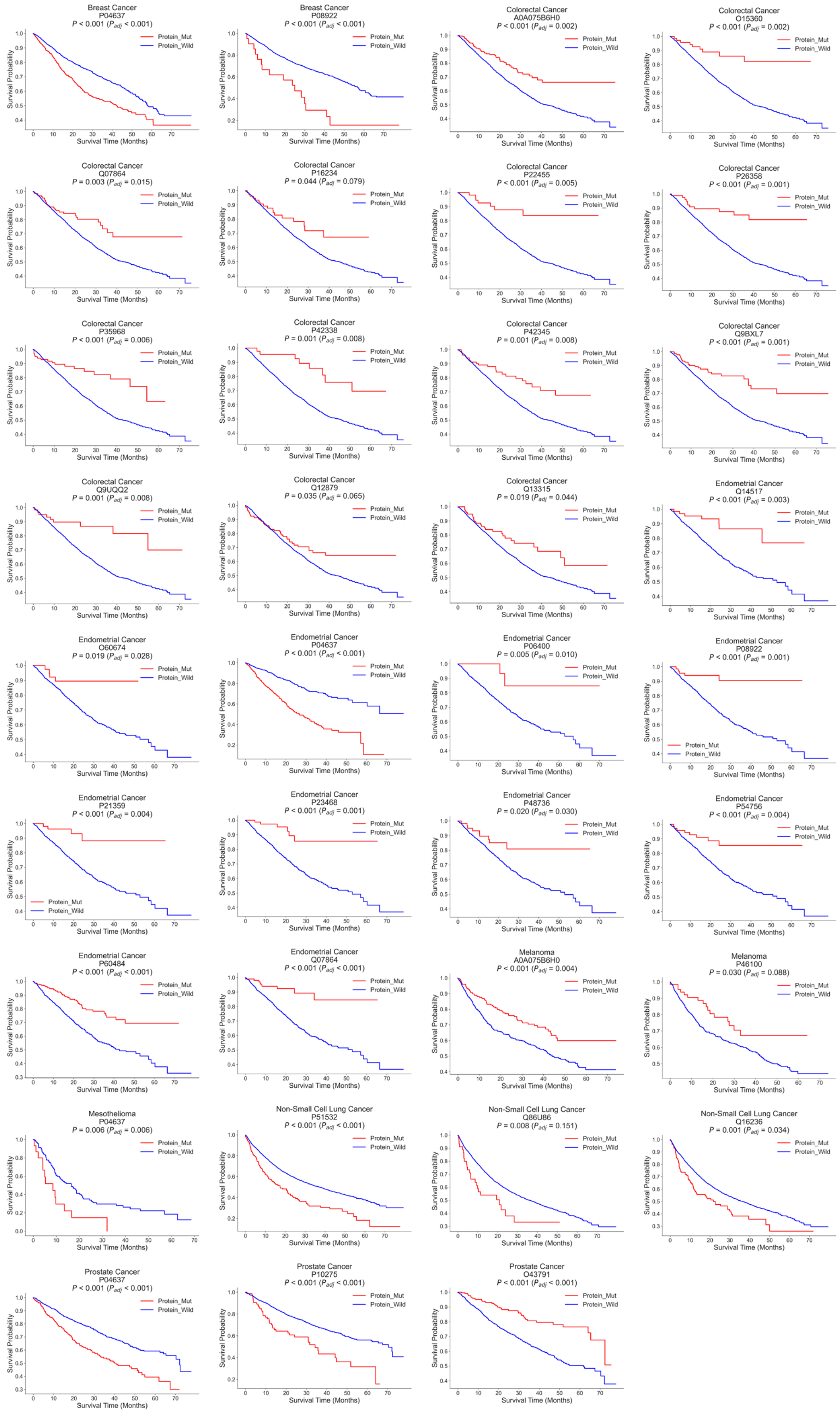

Figure S6. Protein-Wide Mutation–Based Survival Analysis

Patients were stratified based on the presence or absence of any mutations in the target protein. Kaplan-Meier survival curves were used to evaluate the association between protein-level mutation and overall survival. *P*-values were calculated through the Log-Rank test to determine the statistical significance of this association.

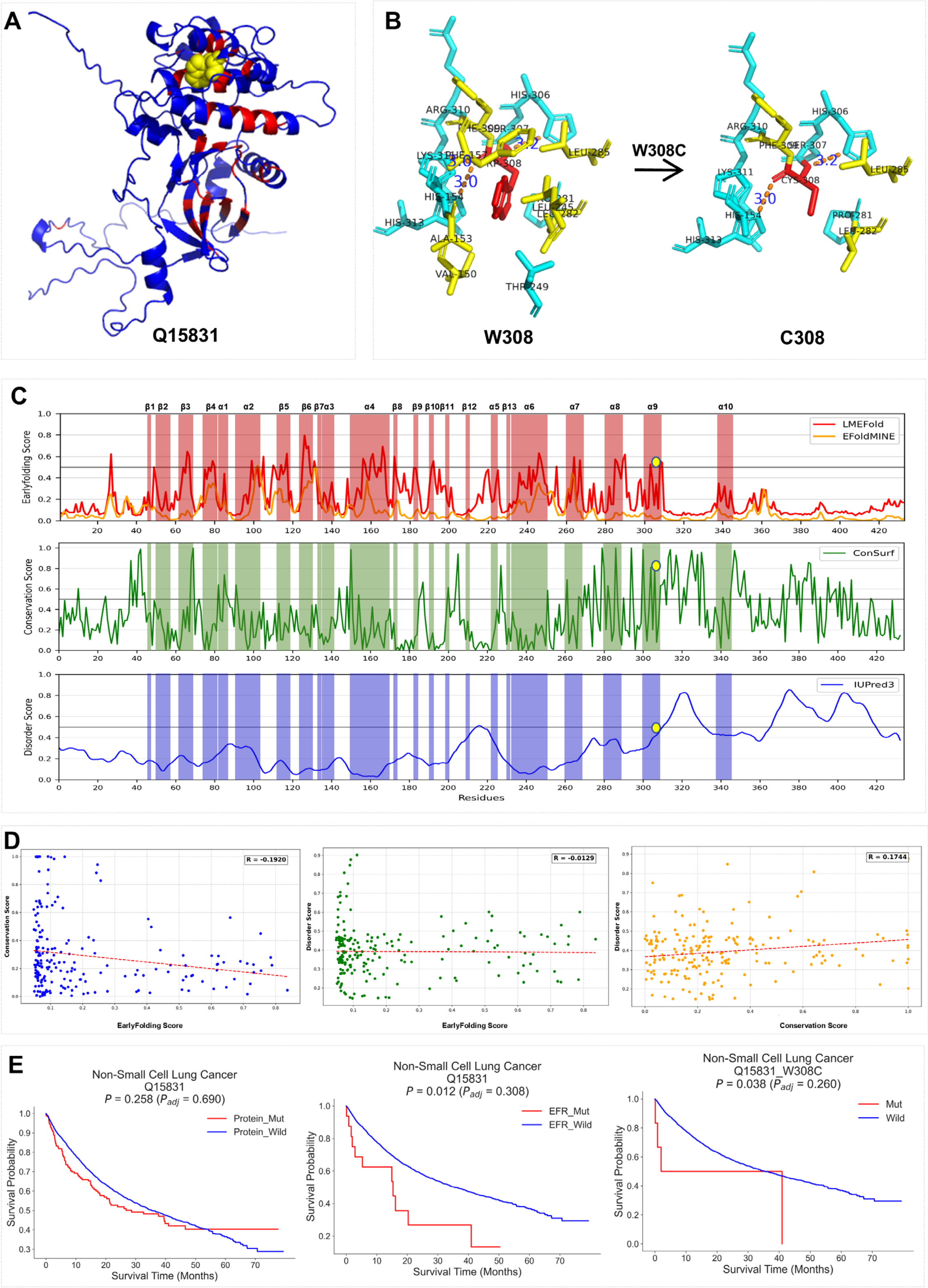

Figure S7. *STK11* early folding prediction, W308 mutation structural impact, and lung cancer survival analysis.

(A) AlphaFold structure of *STK11* (AF-Q15831-F1-model_v4.pdb, corresponds to the canonical UniProt Q15831

sequence); red, high early folding propensity residues; yellow sphere, mutation site W308.

(B) Local comparison of WT *W308* and *C308* mutant: mutant reduces hydrogen bonds, loosens hydrophobic packing, and rearranges structure.

(C) Sequence-level LMEFold early folding scores (red), EFoldMine early folding scores (orange), ConSurf conservation (green), IUPred3 disorder (blue); W308 at α-L folding peak.

(D) Correlation of residue-level early folding scores with conservation and disorder.

(E) MSK-MET lung cancer survival: Overall *STK11* mutation status was not associated with survival (nominal P = 0.258; adjusted P = 0.690). EFR-restricted mutations (nominal P = 0.012; adjusted P = 0.308) and *STK11* p.W308C (nominal P = 0.038; adjusted P = 0.260) showed nominal associations with poorer survival, but neither remained significant after BH correction.

Table S15. Residue numbering mapping between PDB structures and canonical UniProt sequences.

UniProt ID	PDB ID	Region/Segment	PDB Coordinate Range	Canonical Sequence Range
P0CG48	1UBQ	Full-length	1-76	1-76
		β1	2-7	2-7
		β2	12-16	12-16
		α1	23-34	23-34
		β3	41-45	41-45
		β4	48-49	48-49
		3-10 helix	57-59	57-59
		β5	66-71	66-71
P02185	1MBC	Full-length	1-153	1-153
		α1	4-17	4-17
		α2	21-35	21-35
		α3	38-41	38-41
		α4	52-57	52-57
		α5	59-76	59-76
		α6	83-92	83-92
		α7	101-118	101-118
		α8	125-149	125-149
P04637	2XWR_A	Full-length	1-199	91-289
		β1	20-22	110-112
		β2	34-37	124-127
		β3	42-45	132-135
		β4	51-56	141-146
		β5	66-73	156-163
		α1	87-91	177-181
		β6	105-107	195-197
		β7	114-117	204-207
		β8	124-129	214-219
		β9	140-146	230-236
		β10	161-168	251-258

		β11	174-184	264-274
		α2	188-197	278-287
Q15831	AF-Q15831-F1-model_v4	Full-length	1-433	1-433
		β1	45-46	45-46
		β2	49-57	49-57
		β3	61-68	61-68
		β4	74-81	74-81
		α1	82-85	82-85
		α2	91-104	91-104
		β5	113-119	113-119
		β6	124-130	124-130
		β7	134-135	134-135
		α3	136-141	136-141
		α4	150-169	150-169
		β8	172-173	172-173
		β9	182-184	182-184
		β10	190-192	190-192
		β11	199-200	199-200
		β12	209-210	209-210
		α5	222-225	222-225
		β13	231-232	231-232
		α6	233-250	233-250
		α7	260-269	260-269
		α8	280-289	280-289
		α9	300-310	300-310
		α10	338-345	338-345
